# Supplementary material for: Fetal loss and long-term maternal morbidity and mortality: A systematic review and meta-analysis
Source: PLoS Med. 2024 Feb 9;21(2):e1004342. doi: 10.1371/journal.pmed.1004342 (PMC10857720; doi:10.1371/journal.pmed.1004342)
Supplement: S1 Appendix — Fig A in S1 Appendix: Key Word Strategy. Fig B in S1 Appendix: Quality assessment of cohort studies using Adapted Newcastle Ottawa Scale. Fig C in S1 Appendix: Quality assessment of case-controlled studies using Adapted Newcastle Ottawa Scale. Fig D in S1 Appendix: Quality assessment of cross-sectional studies using Adapted Newcastle Ottawa Scale. Fig E in S1 Appendix: Funnel plots of the association of miscarriage and (a) ischaemic heart disease and (b) breast cancer. Fig F in S1 Appendix: Forrest plots presenting pooled RRs for development of (a) ischaemic heart disease, (b) cerebrovascular disease and (c) circulatory diseases for the miscarriage arm using the Mantel–Haenszel random effects model with dichotomous data in meta-analysis. Fig G in S1 Appendix: Forrest plots presenting pooled RRs for development of (a) type 2 diabetes mellitus and (b) depression for the miscarriage arm using the Mantel–Haenszel random effects model with dichotomous data in meta-analysis. Fig H in S1 Appendix: Forrest plots presenting pooled RRs for development of (a) breast cancer, (b) ovarian cancer, and (c) uterine malignancies for the miscarriage arm using the Mantel–Haenszel random effects model with dichotomous data in meta-analysis. Fig I in S1 Appendix: Forrest plots presenting pooled RRs for development of (a) ischaemic heart disease, (b) cerebrovascular disease, (c) breast cancer, and (d) ovarian cancer for recurrent vs. single miscarriage using the Mantel–Haenszel random effects model with dichotomous data in meta-analysis. Fig J in S1 Appendix: Forrest plots presenting pooled RRs for development of (a) ischaemic heart disease, (b) cerebrovascular disease, and (c) breast cancer for recurrent vs. no miscarriage using the Mantel–Haenszel random effects model with dichotomous data in meta-analysis. Fig K in S1 Appendix: Forrest plots presenting pooled RRs for development of (a) ischaemic heart disease, (b) cerebrovascular disease, (c) breast cancer, and (d) ovarian cancer for r [file pmed.1004342.s001.docx]

# Supporting information

Fig A in S1 Appendix: Key Word Strategy

Fig B in S1 Appendix: Quality assessment of cohort studies using Adapted Newcastle Ottawa Scale

Fig C in S1 Appendix: Quality assessment of case-controlled studies using Adapted Newcastle Ottawa Scale

Fig D in S1 Appendix: Quality assessment of cross-sectional studies using Adapted Newcastle Ottawa Scale

Fig E in S1 Appendix: Funnel plots of the association of miscarriage and (a) ischaemic heart disease and (b) breast cancer

Fig F in S1 Appendix: Forrest plots presenting pooled risk ratios (RR) for development of (a) ischaemic heart disease, (b) cerebrovascular disease and (c) circulatory diseases for the miscarriage arm using the Mantel-Haenszel random effects model with dichotomous data in meta-analysis

Fig G in S1 Appendix: Forrest plots presenting pooled risk ratios (RR) for development of (a) breast cancer, (b) ovarian cancer and (c) uterine malignancies for the miscarriage arm using the Mantel-Haenszel random effects model with dichotomous data in meta-analysis

Fig H in S1 Appendix: Forrest plots presenting pooled risk ratios (RR) for development of (a) type 2 diabetes mellitus and (b) depression for the miscarriage arm using the Mantel-Haenszel random effects model with dichotomous data in meta-analysis

Fig I in S1 Appendix: Forrest plots presenting pooled risk ratios (RR) for development of (a) ischaemic heart disease, (b) cerebrovascular disease (c) breast cancer and (d) ovarian cancer for recurrent vs single miscarriage using the Mantel-Haenszel random effects model with dichotomous data in meta-analysis

Fig J in S1 Appendix: Forrest plots presenting pooled risk ratios (RR) for development of (a) ischaemic heart disease, (b) cerebrovascular disease and (c) breast cancer for recurrent vs no miscarriage using the Mantel-Haenszel random effects model with dichotomous data in meta-analysis

Fig K in S1 Appendix: Forrest plots presenting pooled risk ratios (RR) for development of (a) ischaemic heart disease, (b) cerebrovascular disease (c) breast cancer and (d) ovarian cancer for recurrent vs single miscarriage using the Generic inverse variance random effects model meta-analysis

Fig L in S1 Appendix: Forrest plots presenting pooled risk ratios (RR) for development of (a) ischaemic heart disease, (b) cerebrovascular disease and (c) breast cancer for recurrent vs no miscarriage using the Generic inverse variance random effects model with dichotomous data in meta-analysis

Fig M in S1 Appendix: Forrest plots presenting pooled risk ratios (RR) for development of (a) ischaemic heart disease, (b) cerebrovascular disease and (c) circulatory disease for stillbirth arm using the Mantel-Haenszel random effects model with dichotomous data in meta-analysis

Fig N in S1 Appendix: Forrest plots presenting pooled risk ratios (RR) for development of (a) breast cancer, (b) female malignancies and (c) all malignancies for stillbirth arm using the Mantel-Haenszel random effects model with dichotomous data in meta-analysis

Fig O in S1 Appendix: Forrest plots presenting pooled risk ratios (RR) for development of (a) type 2 diabetes, (b) renal disease (c) depression for stillbirth arm using the Mantel-Haenszel random effects model with dichotomous data in meta-analysis

Table A in S1 Appendix: Outcome definitions of the miscarriage arm of the meta-analysis

Table B in S1 Appendix: Outcome definitions of the stillbirth arm of the meta-analysis

Table C in S1 Appendix: Covariate/Confounding variables the researchers adjusted for in the studies of miscarriage.

Table D in S1 Appendix: Covariate/Confounding variables the researchers adjusted for in the studies of stillbirth.

Fig A: Key Word Strategy


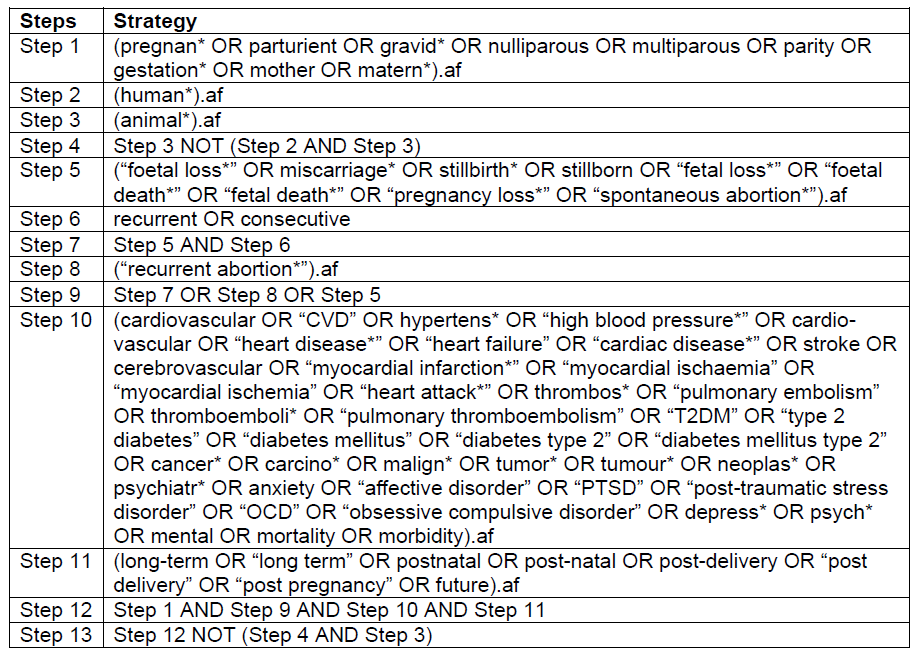


Fig B: Quality assessment of cohort studies using Adapted Newcastle Ottawa Scale

| **Study** | **Adapted NOS quality assessment** | | | | **Risk of Bias** |
| --- | --- | --- | --- | --- | --- |
|  | **Selection** | **Comparability** | **Outcome** | **Total** |  |
| Andalib et al. (2006), Iran [1] | **** | ** | *** | 9/9 | Low |
| Auger et al (2021), Canada [2] | **** | ** | *** | 9/9 | Low |
| Barret et al. (2020,) Sweden [3] | **** | ** | *** | 9/9 | Low |
| *Bergant et al. (1997), Austria* [4] | *** | * | ** | 6/9 | High |
| Bourne et al. (1968), UK [5] | *** | - | *** | 6/9 | High |
| Boyle et a.l (1996), Australia [6] | **** | ** | *** | 9/9 | Low |
| Braem et al. (2012), Netherlands [7] | **** | ** | *** | 9/9 | Low |
| Calderon-Margalit et al. (2007), Israel [8] | *** | ** | *** | 8/9 | Low |
| Charach et al. (2018), Israel [9] | **** | ** | *** | 9/9 | Low |
| Coleman et al. (2013), Denmark [10] | **** | ** | *** | 9/9 | Low |
| Cooper et al. (1999), USA [11] | *** | ** | *** | 8/9 | Low |
| Gallagher et al. (2011), USA [12] | **** | ** | *** | 9/9 | Low |
| Horn et al. (2019), Norway [13] | *** | ** | *** | 8/9 | Low |
| Hvidtjørn et al. (2016), Denmark [14] | **** | ** | *** | 9/9 | Low |
| Janssen et al. (2006), Netherlands [15] | * | - | *** | 4/9 | High |
| Kessous et al. (2014), Israel [16] | **** | ** | *** | 9/9 | Low |
| Kharazmi, Fallah, Luoto (2010), Germany [17] | **** | ** | *** | 9/9 | Low |
| Kharazmi et al. (2012), Germany [18] | **** | ** | *** | 9/9 | Low |
| Kharazmi et al. (2011), Germany [19] | **** | ** | *** | 9/9 | Low |
| Lambalk et al. (2016), Netherlands [20] | **** | ** | *** | 9/9 | Low |
| Okoth et al. (2022), UK [21] | **** | ** | *** | 9/9 | Low |
| Pariente et al. (2014), Israel [22] | **** | ** | *** | 9/9 | Low |
| Parker et al. (2014) USA [23] | **** | ** | *** | 9/9 | Low |
| Peters et al. (2017), China [24] | **** | ** | *** | 9/9 | Low |
| Peters et al. (2019), China [25] | **** | ** | *** | 9/9 | Low |
| Rådestad et al. (1996), Sweden [26] | *** | ** | *** | 9/9 | Low |
| Ranthe et al. (2013), Denmark [27] | **** | ** | *** | 9/9 | Low |
| Schwarzman et al. (2020), Israel [28] | **** | ** | *** | 9/9 | Low |
| Smith et al. (2003) UK [29] | **** | ** | *** | 9/9 | Low |
| Vance et al. (1991), Australia [30] | *** | ** | *** | 8/9 | Low |
| Wagner et al. (2015), Netherlands [31] | **** | ** | *** | 9/9 | Low |
| Yamada et al., (2017), Japan [32] | **** | ** | *** | 9/9 | Low |

Fig C: Quality assessment of case-controlled studies using Adapted Newcastle Ottawa Scale

| **Study** | **Adapted NOS quality assessment** | | | | **Risk of Bias** |
| --- | --- | --- | --- | --- | --- |
|  | **Selection** | **Comparability** | **Outcome** | **Total** |  |
| Adami et al. (1990), Norway [33] | **** | ** | *** | 9/9 | Low |
| Brewster et al. (2005), UK [34] | **** | ** | *** | 9/9 | Low |
| Brinton et al. (1983), USA [35] | **** | ** | ** | 8/9 | Low |
| Bertuccio et al. (2007), Italy [36] | **** | ** | ** | 8/9 | Low |
| Calle et al. (1995), USA [37] | **** | ** | *** | 9/9 | Low |
| Dick et al. (2009), Israel [38] | **** | ** | *** | 9/9 | Low |
| Egerup et al (2020), Denmark [39] | **** | ** | *** | 9/9 | Low |
| Erlandsson et al. (2003), Sweden [40] | **** | ** | *** | 9/9 | Low |
| Goldacre et al. (2001), UK [41] | **** | ** | ** | 8/9 | Low |
| La Vecchia et al. (a) (1987), Italy [42] | **** | ** | *** | 9/9 | Low |
| La Vecchia et al. (b), (1987), Italy [43] | **** | ** | * | 7/9 | Low |
| Laing et al. (1993), USA [44] | **** | * | ** | 7/9 | Low |
| Lipworth et al. (1995), USA [45] | **** | ** | *** | 9/9 | Low |
| Maino et al. (2016), Netherlands [46] | **** | ** | *** | 9/9 | Low |
| Mikkelsen et al. (2019), Denmark [47] | **** | ** | *** | 9/9 | Low |
| Parazzini et al. (1992), Italy [48] | **** | ** | *** | 9/9 | Low |
| Reeves et al. (2006), UK [49] | **** | ** | *** | 9/9 | Low |
| Rosenberg et al. (1988), USA [50] | **** | ** | *** | 9/9 | Low |
| Winkelstein et al. (1958), USA [51] | *** | * | *** | 7/9 | Low |
| Winkelstein, Rekate (1964), USA [52] | *** | * | *** | 7/9 | Low |
| Xu et al. (2004), China [53] | **** | ** | *** | 9/9 | Low |

Fig D: Quality assessment of cross-sectional studies using Adapted Newcastle Ottawa Scale

| **Study** | **Adapted NOS quality assessment** | | | | **Risk of Bias** |
| --- | --- | --- | --- | --- | --- |
|  | **Selection** | **Comparability** | **Outcome** | **Total** |  |
| Kolte et al (2015), Denmark [54] | **** | ** | ** | 8/8 | Low |
| Toffol (a) et al (2013), Finland [55] | *** | ** | ** | 7/8 | Low |
| Toffol (b) et al (2013), Finland [55] | *** | ** | *** | 8/8 | Low |

Fig E: Funnel plots of the association of miscarriage and (a) ischaemic heart disease and (b) breast cancer


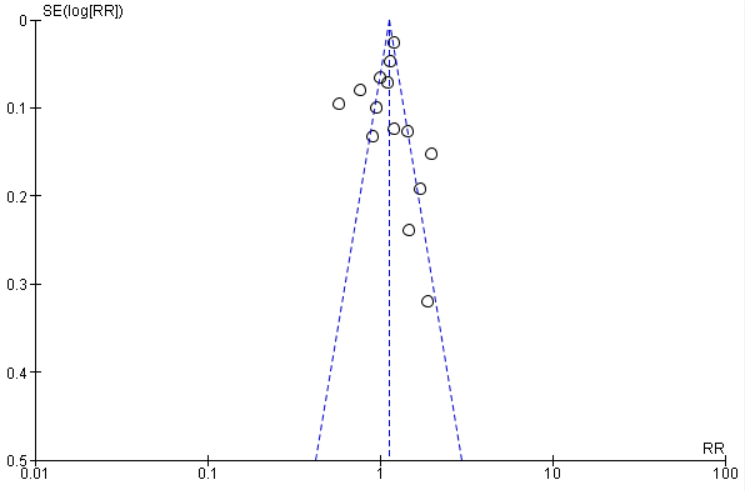
(a)


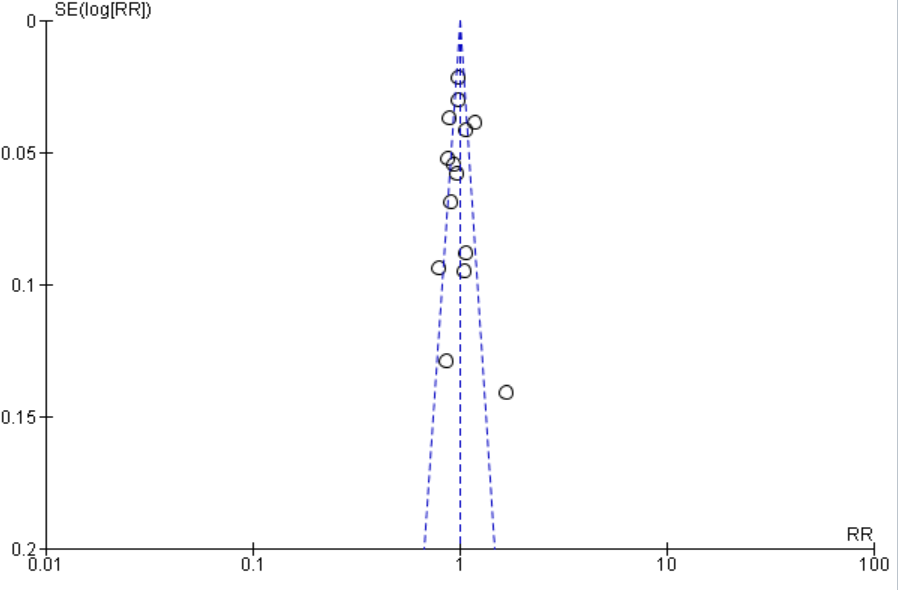
(b)

*RR; Risk Ratio, SE; Standard Error*

Fig F: Forrest plots presenting pooled risk ratios (RR) for development of (a) ischaemic heart disease, (b) cerebrovascular disease and (c) circulatory diseases for the miscarriage arm using the Mantel-Haenszel random effects model with dichotomous data in meta-analysis

(a)


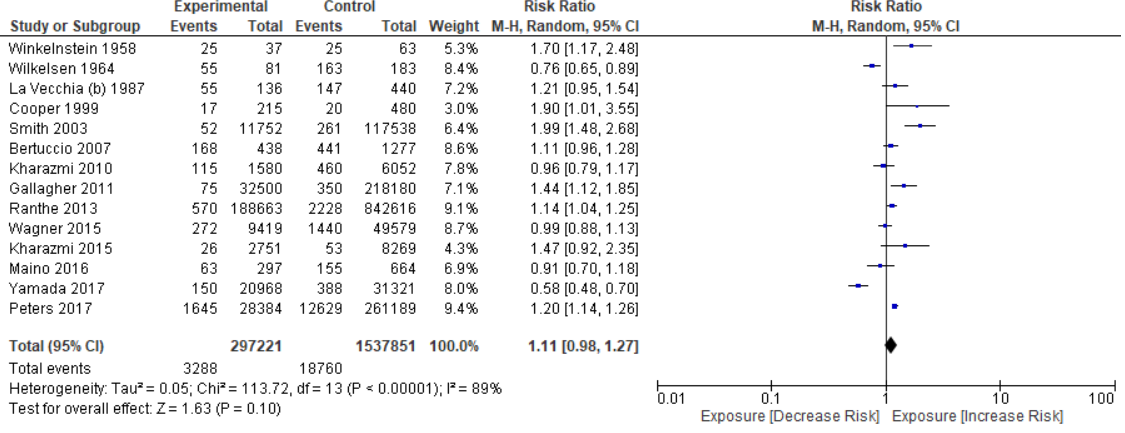


(b)


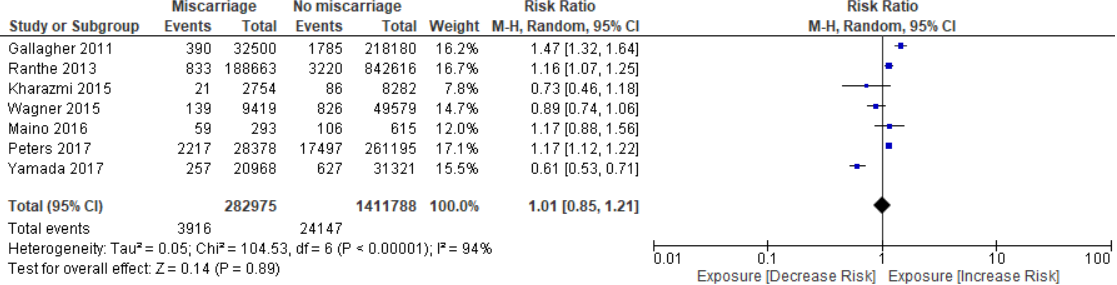


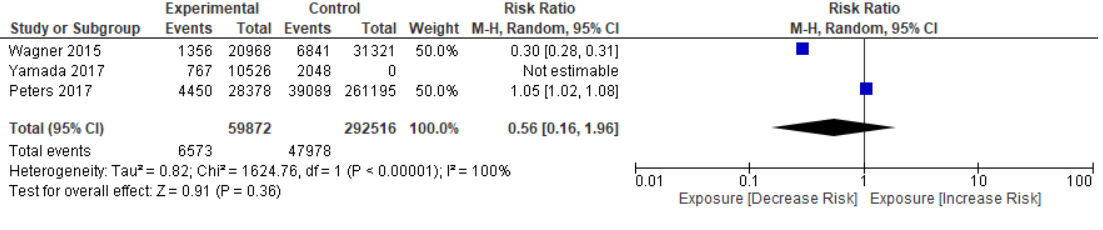
(c)

*CI; Confidence Interval, M-H; Mantel-Haenszel*

Fig G: Forrest plots presenting pooled risk ratios (RR) for development of (a) breast cancer, (b) ovarian cancer and (c) uterine malignancies for the miscarriage arm using the Mantel-Haenszel random effects model with dichotomous data in meta-analysis

(a)


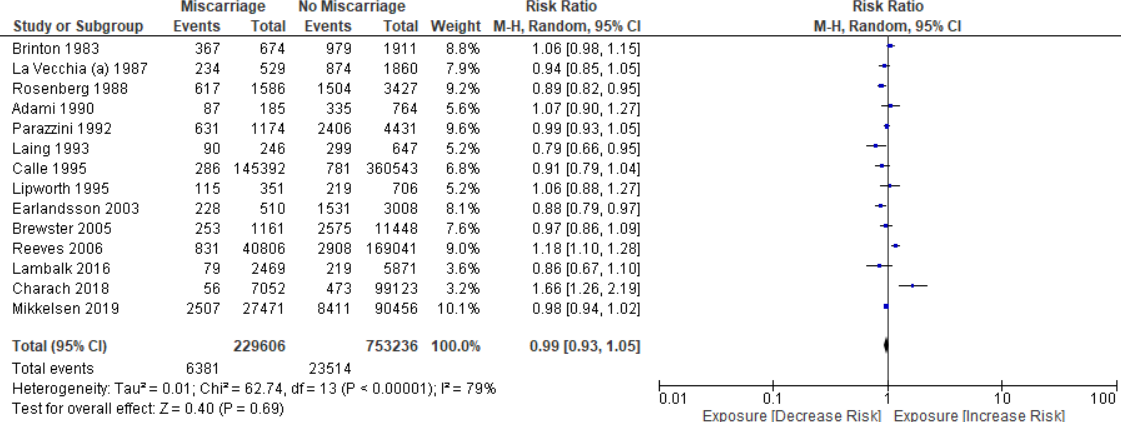


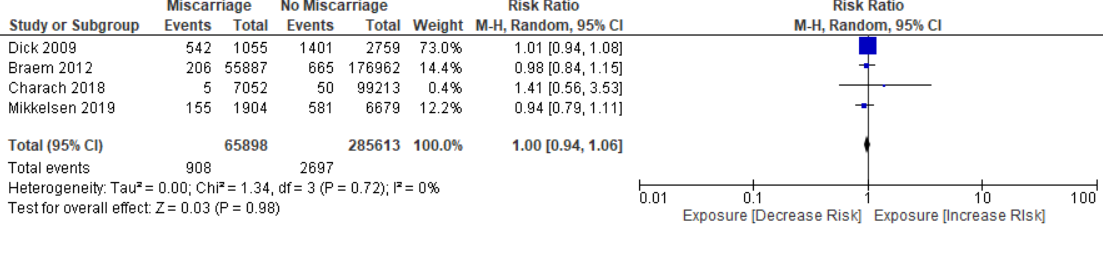
(b)

(c)


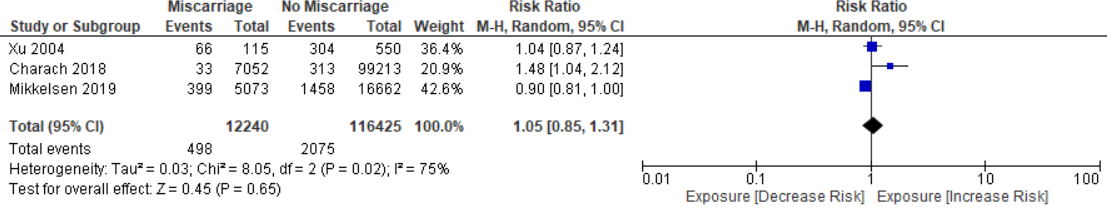


*CI; Confidence Interval, M-H; Mantel-Haenszel*

Fig H: Forrest plots presenting pooled risk ratios (RR) for development of (a) type 2 diabetes mellitus and (b) depression for the miscarriage arm using the Mantel-Haenszel random effects model with dichotomous data in meta-analysis

(a)


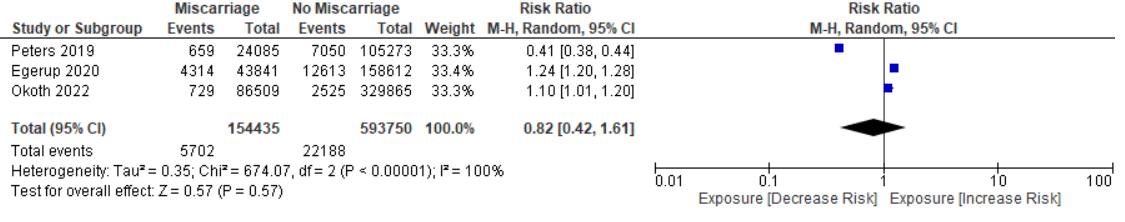


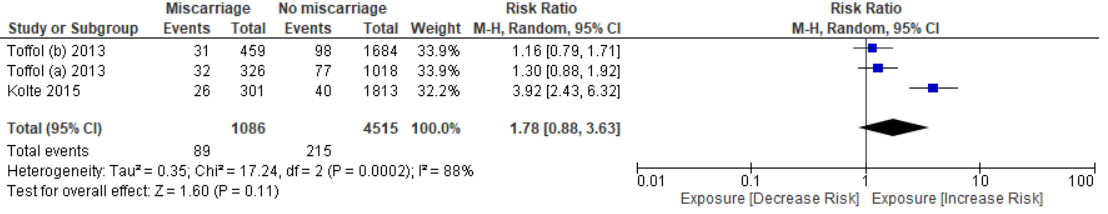
(b)

*CI; Confidence Interval, M-H; Mantel-Haenszel*

Fig I: Forrest plots presenting pooled risk ratios (RR) for development of (a) ischaemic heart disease, (b) cerebrovascular disease (c) breast cancer and (d) ovarian cancer for recurrent vs single miscarriage using the Mantel-Haenszel random effects model with dichotomous data in meta-analysis


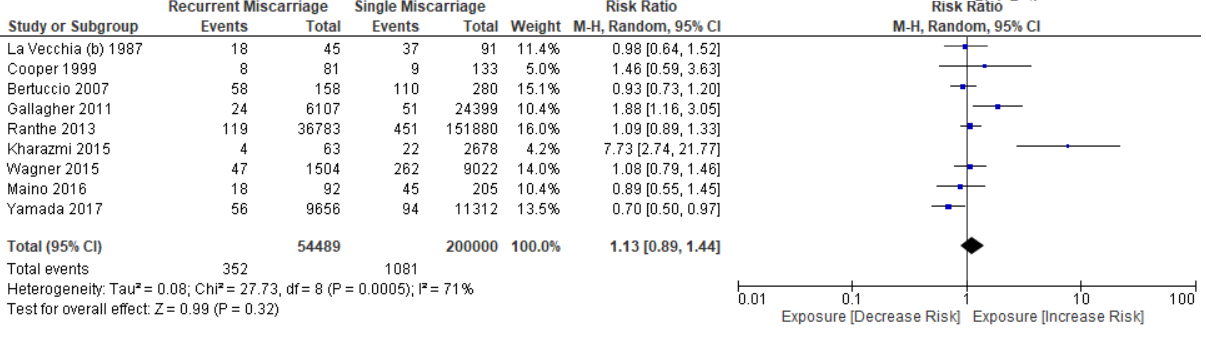
(a)

(b)


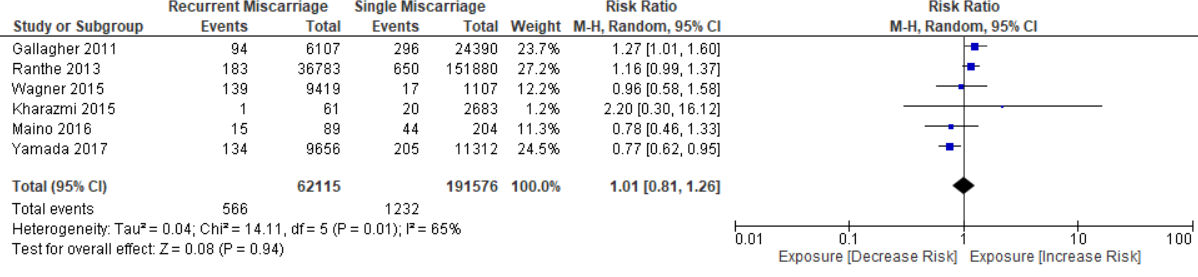


(c)


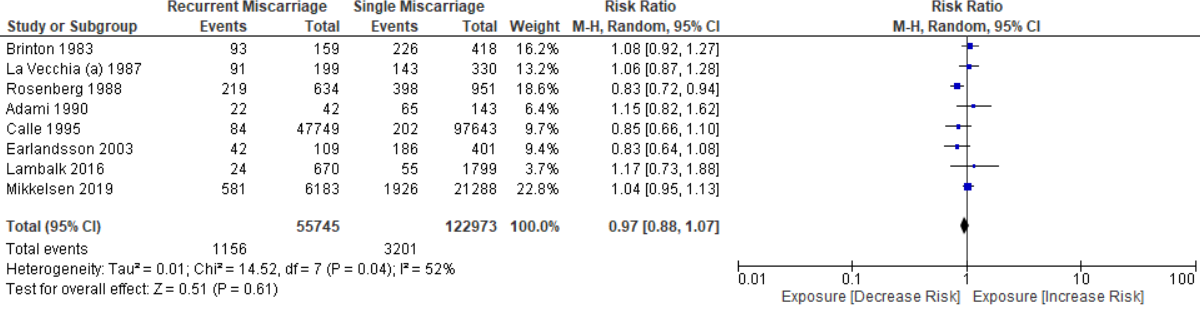


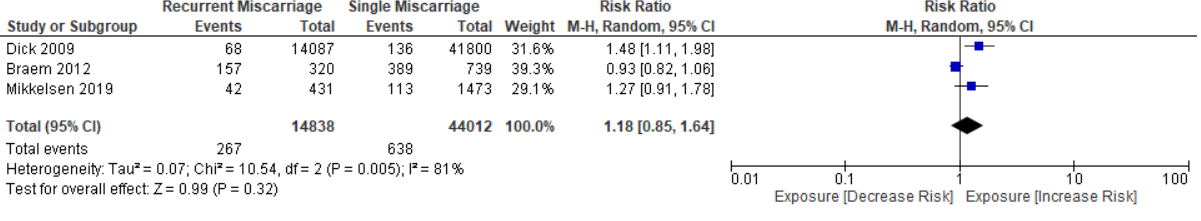
(d)

*CI; Confidence Interval, M-H; Mantel-Haenszel*

Fig J: Forrest plots presenting pooled risk ratios (RR) for development of (a) ischaemic heart disease, (b) cerebrovascular disease and (c) breast cancer for recurrent vs no miscarriage using the Mantel-Haenszel random effects model with dichotomous data in meta-analysis


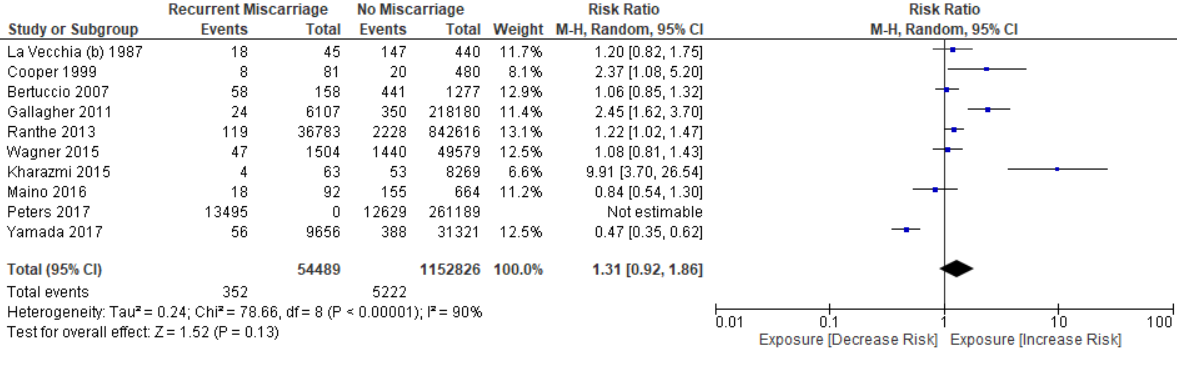
(a)

(b)


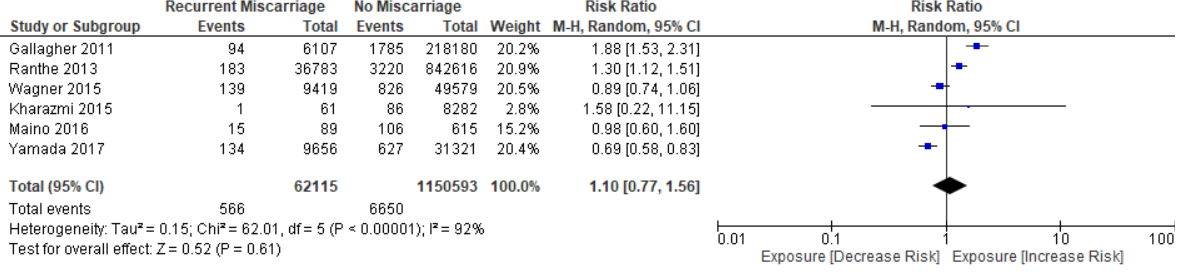


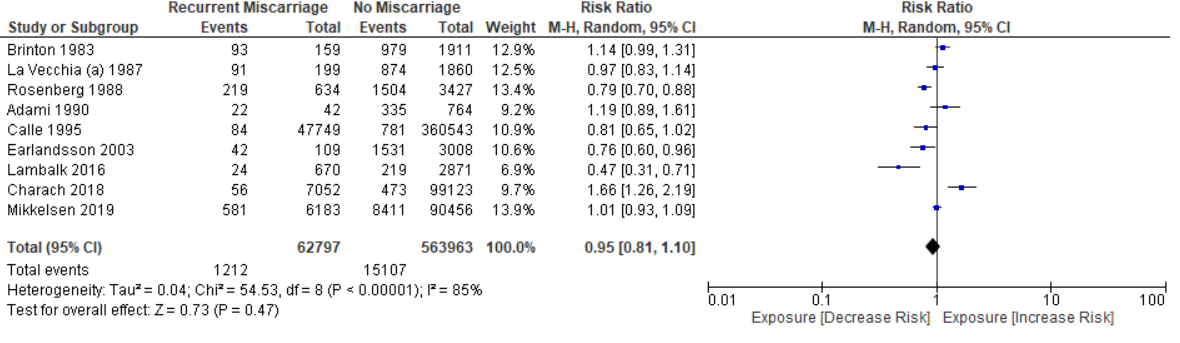
(c)

*CI; Confidence Interval, M-H; Mantel-Haenszel*

Fig K: Forrest plots presenting pooled risk ratios (RR) for development of (a) ischaemic heart disease, (b) cerebrovascular disease (c) breast cancer and (d) ovarian cancer for recurrent vs single miscarriage using the Generic inverse variance random effects model meta-analysis


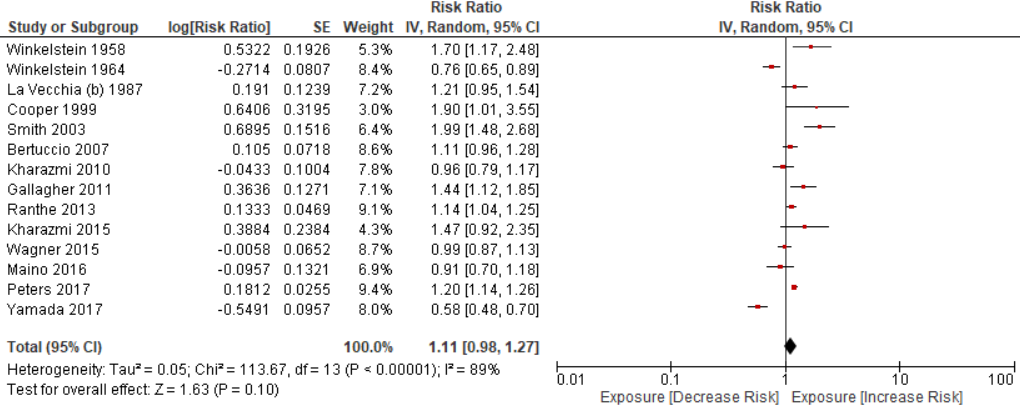
(a)


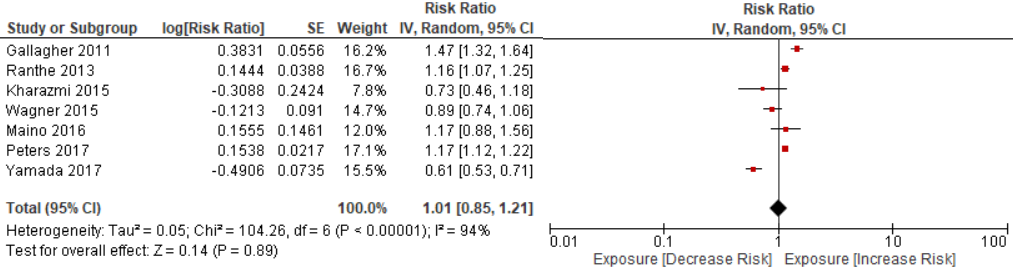
(b)


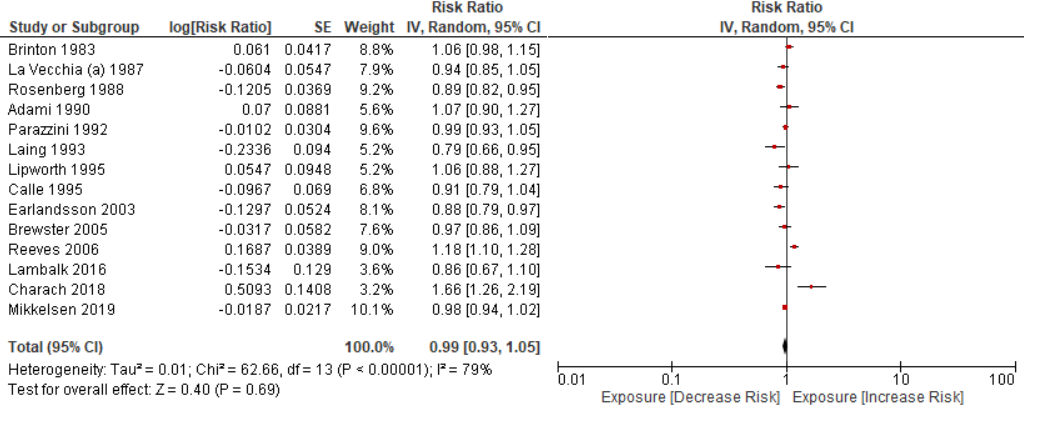
(c)


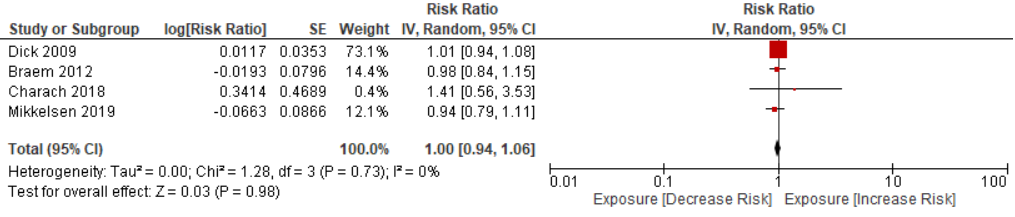
(d)

*CI; Confidence Interval, M-H; Mantel-Haenszel*

Fig L: Forrest plots presenting pooled risk ratios (RR) for development of (a) ischaemic heart disease, (b) cerebrovascular disease and (c) breast cancer for recurrent vs no miscarriage using the Generic inverse variance random effects model with dichotomous data in meta-analysis

(a)


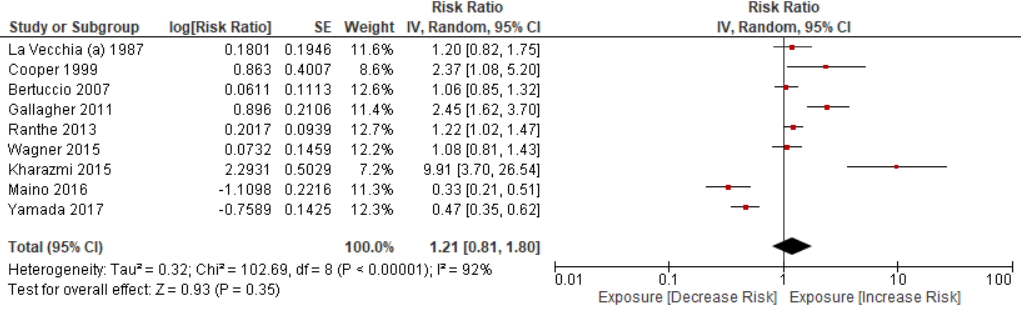


(b)


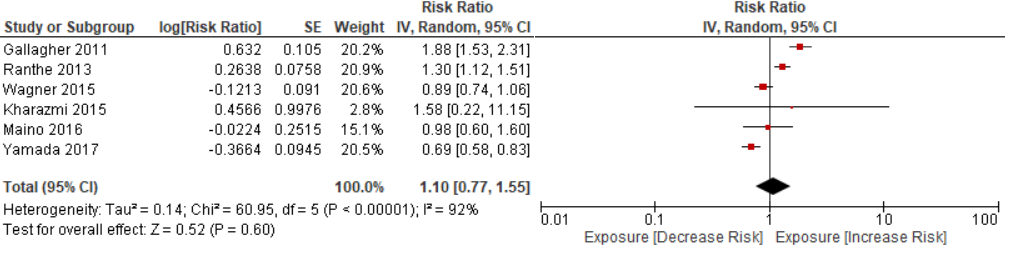


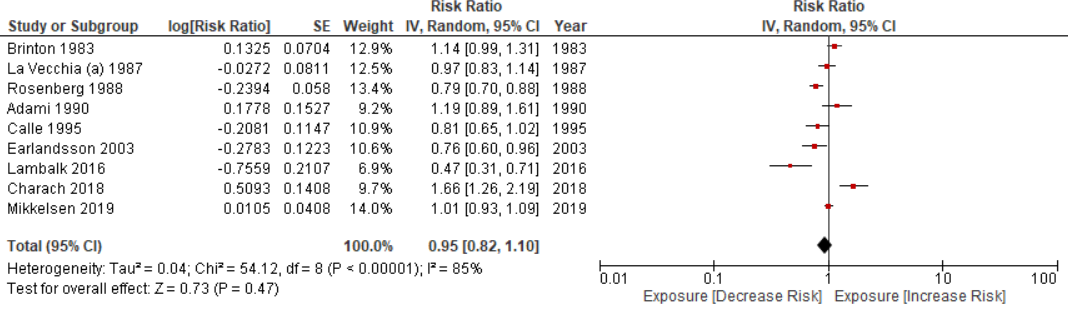
(c)

*CI; Confidence Interval, M-H; Mantel-Haenszel*

Fig M: Forrest plots presenting pooled risk ratios (RR) for development of (a) ischaemic heart disease, (b) cerebrovascular disease and (c) circulatory disease for stillbirth arm using the Mantel-Haenszel random effects model with dichotomous data in meta-analysis

(a)


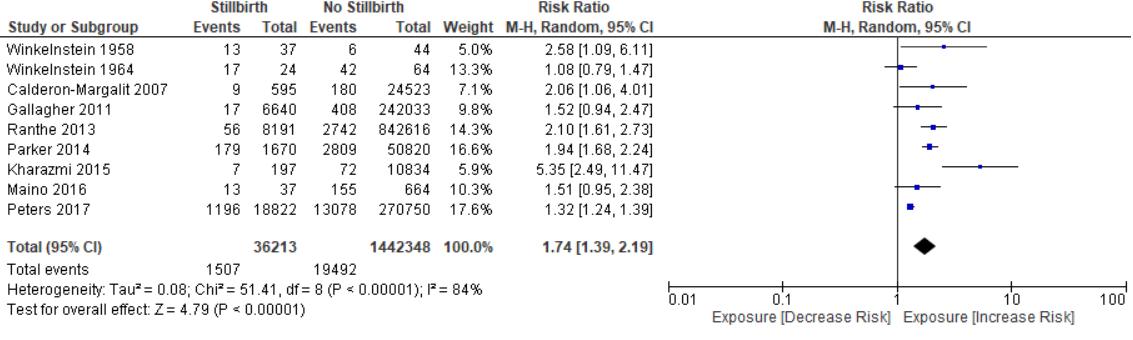


(b)


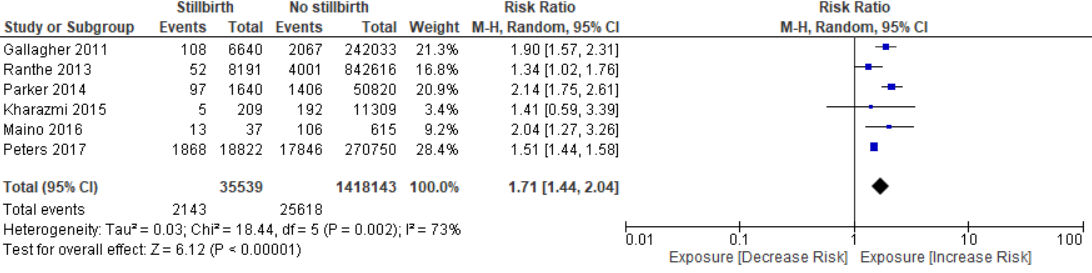


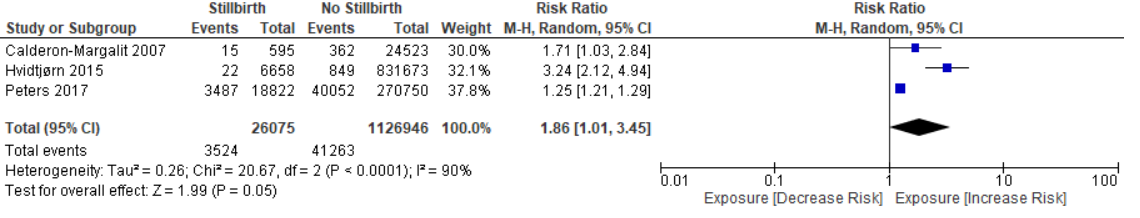
(c)

*CI; Confidence Interval, M-H; Mantel-Haenszel*

Fig N: Forrest plots presenting pooled risk ratios (RR) for development of (a) breast cancer, (b) female malignancies and (c) all malignancies for stillbirth arm using the Mantel-Haenszel random effects model with dichotomous data in meta-analysis

(a)


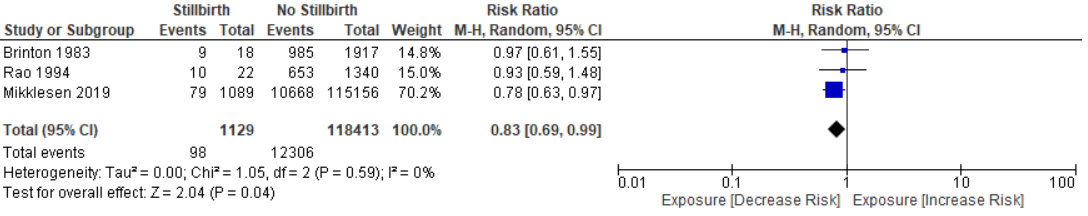


(b)


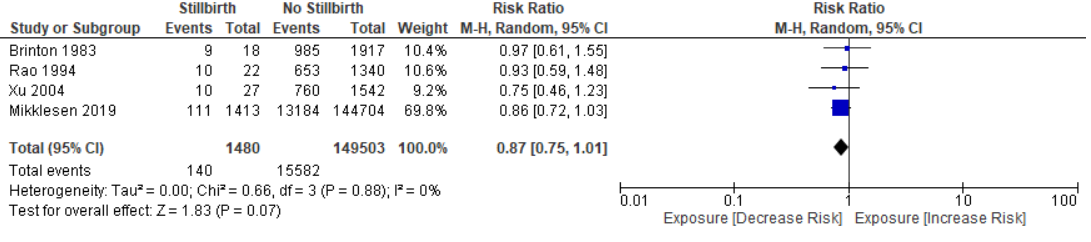


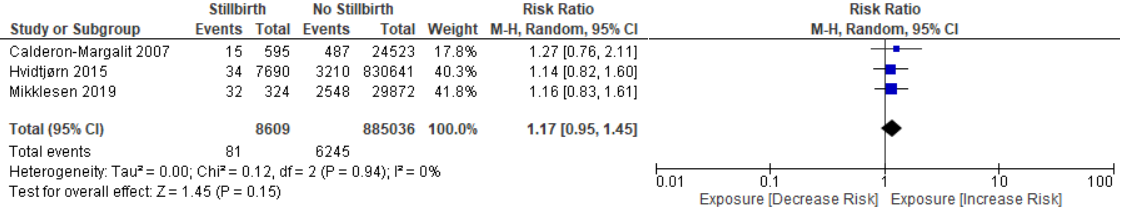
(c)

*CI; Confidence Interval, M-H; Mantel-Haenszel*

Fig O: Forrest plots presenting pooled risk ratios (RR) for development of (a) type 2 diabetes, (b) renal disease (c) depression for stillbirth arm using the Mantel-Haenszel random effects model with dichotomous data in meta-analysis

(a)


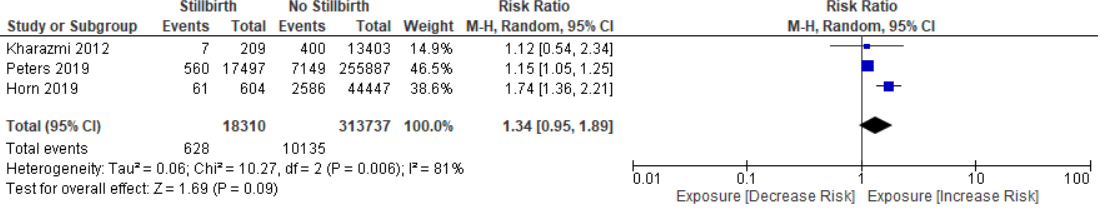


(b)


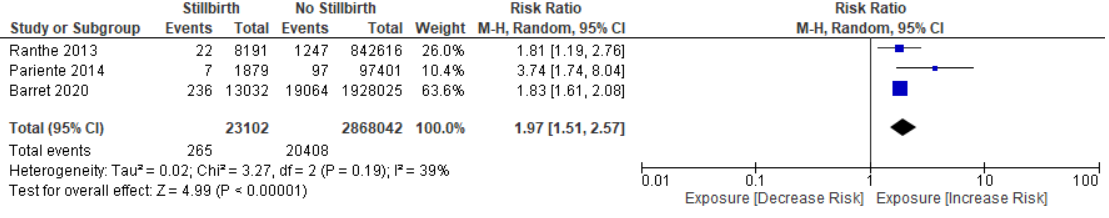


(c)


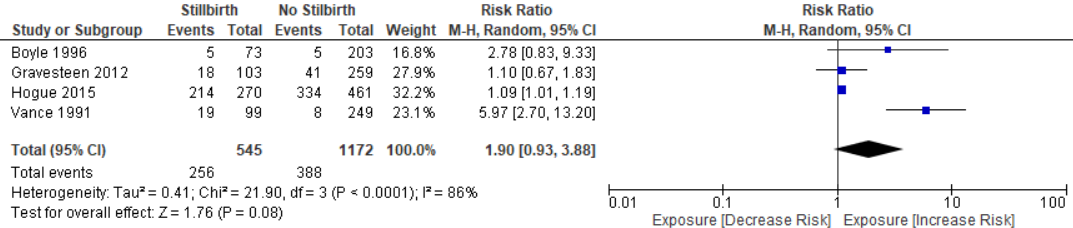


*CI; Confidence Interval, M-H; Mantel-Haenszel*

Table A: Outcome definitions of the miscarriage arm of the meta-analysis

| ***Lead author and publication date (Country)*** | **Outcome** | **Outcome Definition** |
| --- | --- | --- |
| *Adami et al. (1990), Norway* [33] | Breast Cancer | Breast cancer: hospital records of histologically confirmed invasive breast cancer |
| *Andalib et al. (2006), Iran* [1] | Stress | Stress: Holmes and Rahe social re-adjustment rating scale |
|  | Depression | Depression: Beck Depression Inventory |
| *Auger et al (2021), Canada* [2] | Mortality | Mortality: Record linkage |
| *Bergant et al. (1997), Austria* [4] | Anxiety | Anxiety: State–Trait–Anxiety Inventory |
|  | Somatisation | Somatization of symptoms: Giessener Beschwerdebogen |
|  | Life satisfaction | Life Satisfaction Questionnaire |
|  | Depression | Depression: Beck Depression Inventory |
| *Bertuccio et al. (2007), Italy* [36] | Acute Myocardial Infarction | Clinical diagnosis |
| *Braem et al. (2012), Netherlands* [7] | Ovarian, fallopian tube and primary peritoneal cancer | (ICD-O-2 codes C56.9, C57.0, C48) |
| *Brewster et al. (2005), UK* [34] | Breast Cancer | Breast cancer: Record linkage from hospital records and death recrods |
| *Brinton et al. (1983), USA* [35] | Breast Cancer | Breast cancer: detected from clinical records based on clinical examination, mammography, thermography |
| *Calle et al. (1995), USA* [37] | Breast Cancer | Breast cancer deaths (ICD-9, codes 174.0-174.9) |
| *Charach et al. (2018), Israel* [9] | Ovarian Cancer | Ovarian cancer: record linkage based on ICD-9 |
|  | Uterine Cancer | Uterine cancer: record linkage based on ICD-9 |
|  | Breast Cancer | Cervix cancer: record linkage based on ICD-9 |
| *Coleman et al. (2013), USA* [10] | Mortality | All-cause mortality |
| *Cooper et al. (1999), USA* [11] | IHD | Self-diagnosis |
| *Dick et al. (2009), Israel* [38] | Ovarian Cancer | Ovarian cancer: Histologically confirmed primary epithelial ovarian cancer from hospital records |
| *Egerup et al (2020), Denmark* [39] | T2DM | Classification of Diseases (ICD); ICD-8 from 1977 to 1994 and ICD-10 from 1994onward |
| *Erlandsson et al. (2003), Sweden* [40] | Breast Cancer | Breast cancer: Primary breast cancer diagnosis (linkage records) between 1973-1991) |
| *Gallagher et al. (2011), USA* [12] | Death from IHD | Death from ischaemic heart disease: Retrieved from population registries based on ICD-9 |
|  | Death from IS | Death from ischaemic stroke: Retrieved from population registries based on ICD-9 |
|  | Death from HS | Death from Haemorrhagic stroke: Record retrieval from population registries based on ICD-9 |
| *Goldacre et al. (2001), UK* [41] | Breast Cancer | Breast cancer: Primary breast cancer diagnosis (linkage records) |
| *Horn et al. (a) (2019), Norway* [13] | Hypertension | Physician diagnosed |
|  | Hypercholesterolaemia | Hypercholesterolaemia: physician diagnosed or report of cholesterol lowering medication |
|  | T2DM | Type 2 diabetes: records denoting T2D diagnosis based on the 1997 American Diabetes Association criteria |
| *Horn et al. (b) (2019), Norway* [13] | Hypertension | Physician diagnosed |
|  | Hypercholesterolaemia | Hypercholesterolaemia: physician diagnosed or report of cholesterol lowering medication |
|  | T2DM | Type 2 diabetes: records denoting T2D diagnosis based on the 1997 American Diabetes Association criteria |
| *Janssen et al. (2006), Netherlands* [15] | Anxiety, Depression, Somatisation, OCD: SCL-90 | - |
| *Kessous et al. (2014), Israel* [16] | Non-invasive cardiovascular diagnostic procedures: Hospitalization based on ICD-9 | Non-invasive cardiovascular diagnostic procedures: Hospitalization based on ICD-9 |
|  | Cardiac invasive diagnostic procedures: Hospitalization based on ICD-9 | Cardiac invasive diagnostic procedures: Hospitalization based on ICD-9 |
| *Kharazmi et al. (2011), Germany* [19] | MI | Myocardial infarction: ICD-10, codes: I12.0, I21.4 |
|  | Stroke | Stroke: ICD-10, codes: I60.0-I162.9 |
| *Kharazmi et al. (2012), Germany* [18] | T2DM | Type 2 Diabetes: ICD-10, codes E10, E11 |
| *Kharazmi, Fallah, Luoto (2010), Germany* [17] | Hypertension | Hypertension: Assessed by record linkage, drug history and |
|  | Angina Pectoris | Angina pectoris: Assessed by record linkage, drug history and physicians examination |
| *Kolte et al. (2015), Denmark* [54] | Stress: Cohen’s Perceived Stress Scale based on ICD-10 | Stress: Cohen’s Perceived Stress Scale based on ICD-10 |
|  | Depression | - |
| *La Vecchia et al. (a) (1987), Italy* [42] | Breast cancer | Breast cancer: Histologically confirmed breast cancer |
| *La Vecchia et al. (b), (1987), Italy* [43] | MI | Myocardial infarction: Record linkage based on the standard World Health Organization criteria |
| *Laing et al. (1993), USA* [44] | Breast cancer | Histologically confirmed cancer from medical records and cancer registry |
| *Lambalk et al. (2016), Netherlands* [20] | Breast cancer | Record linkage |
| *Lipworth et al. (1995), USA* [45] | Breast cancer | Histologically confirmed breast cancer from medical records, cancer registry |
| *Maino et al. (2016), Netherlands* [46] | MI | Myocardial infarction: presence of clinical symptoms |
|  | IS | Ischaemic stroke: clinical storke was confirmed by either CT or MRI |
|  | Any arterial thrombosis |  |
| *Mikkelsen et al. (2019), Denmark* [47] | Breast cancer | Breast cancer: national registery record linkage identification |
|  | Ovarian cancer | Ovarian cancer: national registry linkage identification |
|  | Endometrial cancer | Endometrial Cancer |
|  | Cervical Cancer | Cervical cancer: national record linkage |
|  | Bladder cancer | Bladder cancer |
|  | Renal cancer | Renal cancer |
|  | Lung cancer | Lung cancer |
|  | GI cancer | GI cancer |
|  | Brain cancer | Brain cancer |
|  | Haematological cancer | Haematological cancer |
|  | Melanoma | Melanoma |
|  | All cancers mortality | All cancers mortality |
| *Okoth et al (2022), UK* [21] | Hypertension | Incidental diagnosis |
|  | T2DM | Incidental diagnosis |
| *Parazzini et al. (1992), Italy* [48] | Breast cancer | Breast cancer: histologically confirmed |
| *Pell, Smith, Walsh (2003), UK* [29] | Cerebrovascular events | Ischaemic stroke, Intracranial haemorrhage, TIA: Death or hospital admission due to a principal diagnosis of cerebrovascular disease (ICD-9), codes 430–438, ICD-10 Codes 160-169 & G45 |
| *Peters et al. (2017), UK* [24] | CHD | Coronary heart disease: ICD-10 (I20–I25) |
|  | Stroke | Stroke: ICD-10 I60–I61, I63–I64 |
|  | All circulatory diseases | All circulatory diseases: ICD-10 I00-I99 |
| *Peters et al. (2020), UK* [25] | Incident T2DM | Incident Type 2 diabetes: ICD-10, codes E10-E14 |
| *Ranthe et al. (2013), Denmark* [27] | MI | Myocardial infarction: (ICD-8 code 410 and ICD-10 codes I21–I22) |
|  | Cerebral infarction | Cerebral infarction: ICD-8 code 436 and ICD-10 codes I63–I64 |
|  | Renovascular hypertension | Renovascular hypertension: (ICD-8 code 400.39 and ICD-10 codes I12–I13 and I15.0) |
| *Reeves et al. (2006), UK* [49] | Invasive breast cancer | Incident invasive breast cancer |
| *Rosenberg et al. (1988), USA* [50] | Breast cancer | Record linkage |
| *Schwarzman et al. (2020), Israel* [28] | Arterial thromboembolic events: | Arterial thromboembolic events: record linkage and ICD-9 |
|  | Venous thromboembolic events | Venous Thromboembolic events: Record linkage and ICD-9 |
| *Smith et al. (2003) UK* [56] | Death from IHD | Death from ischaemic heart disease: ICD-9, codes (410-414) or ICD-10, Codes (I20-I25) |
| *Toffol, Koponen, Partonen (a) (2013), Finland* [55] | Alcohol abuse | Composite International Diagnostic Interview (CID) based on DSM-IV |
|  | Alcohol dependence | Composite International Diagnostic Interview (CID) based on DSM-IV |
|  | Dysthymic disorder | Composite International Diagnostic Interview (CID) based on DSM-IV |
|  | Major Depressive episode | A modified version for the Finnish population of the Beck Depression Inventory (BDI) |
|  | Anxiety disorder | Composite International Diagnostic Interview (CID) based on DSM-IV |
| *Toffol et al. (b) (2013), Finland* [55] | Depressive symptoms |  |
|  | Recent depression diagnosis | Recent depression diagnosis: n the previous month they had had one or more out of 13 |
|  | Recent psychiatric diagnosis | Recent psychiatric diagnosis: non- structured questionnaire,where the participants were asked how often in the previous month they had had one or more out of 13 somatic and psychological symptoms |
|  | Anhedonia during the last year | Anhedonia during the last year: non- structured questionnaire, where the participants were asked how often in the previous month they had had one or more out of 13 somatic and psychological symptoms |
| *Wagner et al. (2015), Netherlands* [31] | IHD | Ischaemic heart disease: Hospital & death records based on ICD-9 411, 413-414, ICD-10 120-125 |
|  | Cerebrovascular disease | Cerebrovascular disease: Record linkage based on ICD-9 430-438, ICD10-160-169, G45 |
|  | Composite cardiovascular outcome | Composite cardiovascular outcome: admission to hospital or death due to diseases of the circulatory system (ICD-9 390-459, ICD-10 100-199, G45) or cardiac surgery (OPCS54 Classification of Surgical Operations and Procedures: K40 saphenous vein graft replacement of coronary artery, K51 diagnostic transmural operations on coronary artery, K65 catheterization of heart, K75 percutaneous transluminal balloon angioplasty and insertion of stent into coronary artery |
| *Winkelstein et al. (1958), USA* [51] | Myocardial infarction | Myocardial infarction: Characteristic history of myocardial infarction, positive ECG findings, and had to have survived for the interview |
| *Winkelstein, Rekate (1964), USA* [52] | Atherosclerotic Heart Disease | Atherosclerotic heart disease: Hospital record retrieval based on ICD 420.0, 420.1, 420.2 |
| *Xu et al. (2004), China* [53] | Endometrial cancer | Endometrial cancer: record linkage from histologically confirmed cancer |
| *Yamada et al., (2017), Japan* [32] | Death from Stroke (total) | Death from Stroke (total): ICD - 10 codes: I60-69, I20-25, I01-I99 |
|  | Death from IS | Death from ischaemic stroke: ICD - 10 codes: I60-69, I20-25, I01-I99 |
|  | Death from haemorrhagic stroke | Death from Haemorrhagic stroke: ICD - 10 codes: I60-69, I20-25, I01-I99 |
|  | Death from intracranial haemorrhage | Death from intracranial haemorrhage: ICD - 10 codes: I60-69, I20-25, I01-I99 |
|  | Subarachnoid haemorrhage | Subarachnoid haemorrhage: ICD - 10 codes: I60-69, I20-25, I01-I99 |
|  | Death from CHD | Death from Coronary heart disease: Confidence interval lower limit outcome 5 (2 losses) |
|  | Total cardiovascular disease | Total cardiovascular disease: ICD - 10 codes: I60-69, I20-25, I01-I99 |

*CHD; Coronary Heart Disease, CT; Computerised Tomography, DSM; Diagnostic and Statistical Manual of Mental Disorders, GI; Gastrointestinal, HS; Haemorrahic Stroke, ICD; International Classification of Diseases, IHD; Ishaemic Heart Disease, IS; Ishaemic Stroke, MI; Myocardial Infarction, MRI; Magnetic Resonance Imaging, OCD; Obsessive Compulsive Disorder, T2DM, Type 2 Diabetes Mellitus, TIA; Transient Ischaemic Attack*

Table B: Outcome definitions of the stillbirth arm of the meta-analysis

| *Lead author and publication date (Country)* | Outcome | Outcome Definition |
| --- | --- | --- |
| *Auger et al (2021), Canada* [2] | Mortality | Mortality: record linkage |
| *Barret et al. (2020), Sweden* [3] | CKD | Chronic Kidney disease (CKD) defined with ICD10 codes |
|  | ESRF | End stage renal failure defined as stage 5 CKD requiring dialysis or renal transplantation |
| *Bourne et al. (1968), UK* [5] | Any type of psychological symptoms | Psychological symptoms of any kind: self-reported |
| *Boyle et al (1996), Australia* [6] | Delusion Symptoms | Depression: Delusions Symptoms States Inventory: self-report |
|  |  | Anxiety: Delusions Symptoms States Inventory |
| *Brinton, Hoover, Fraumeni, (1983), USA* [35] | Breast Cancer | Breast cancer: Detected from clinical records based on clinical examination, mammography, thermography |
| *Calderon-Margalit et al. (2007), Israel* [8] | CHD | Coronary heart disease (from 1978-1997 based on ICD-9 and from 1997-1998 based ICD-10 |
|  | Circulatory Disease | Circulatory diseases (from 1978-1997 based ICD-9 and from 1997-1998 on ICD-10) |
|  | Mortality from cancers | Mortality from all neoplasms: from 1978-1997 based on ICD-9 codes and from 1997-1998 based on ICD-10 |
| *Calle et al. (1995), USA* [37] | Breast Cancer | Breast cancer: Deaths from clinical records based on ICD-9, codes 174.0-174.9) |
| *Coleman et al. (2013), Denmark* [10] | All-causes mortality | Mortality: record linkage |
| *Egerup et al (2020), Denmark* [39] | Type 2 Diabetes | Classification of Diseases (ICD); ICD-8 from 1977 to 1994 and ICD-10 from 1994onward |
| *Gallagher et al. (2011), USA* [12] | IHD | Death from ischaemic heart disease: record linkage based on ICD-9 |
|  | IS | Death from ischaemic stroke: record linkage based on ICD-9 |
|  | HS | Death from haemorrhagic stroke: record linkage based on ICD-9 |
| *Gravesteen et al. (2012), Norway* [57] | Depression | Depression: Centre of Epidemiological Studies |
|  |  | Wellbeing: General Health Questionnaire (GHQ-20) |
|  |  | Self-reported quality of life: Ferrans and Powers QOL Index |
| *Hogue et al. (2015), USA* [58] | Depression | Depression: Edinburgh Depression Scale (EDS) --> a score of >12: patient interview |
| *Horn et al. (2019), Norway* [13] | HTN | Hypertension: physician diagnosed |
|  | HC | Hypercholesterolaemia: Physician diagnosed or report of cholesterol lowering medication |
|  | T2DM | Type 2 diabetes: records recording T2D diagnosis based on the 1997 American Diabetes Association criteria |
| *Hvidtjørn et al. (2016), Denmark* [14] | Circulatory Mortality | Death due to cardiovascular disease including all circulatory diseases and coronary heart disease based on ICD8 3900-4299 and ICD10 I00-99 |
|  |  | Death from any type of cancer based on ICD8 1400-2099 and ICD10 C00-99 |
|  | Cancer Mortality | Death from any renal disease based on: ICD8 5800-5999 and ICD10 N00-N39 |
|  |  | Overall mortality |
|  | Renal Mortality | Mortality from other natural causes |
|  |  | All natural causes |
| *Kharazmi et al. (2011), Germany* [19] | MI | Myocardial infarction: ICD-10, codes: I12.0, I21.4 |
|  | Stroke | Stroke: ICD-10, codes: I60.0-I162.9 |
| *Kharazmi et al. (2012), Germany* [18] | T2DM | Type 2 Diabetes: ICD-10, codes: E10, E11, E14 |
| *Maino et al. (2016), Netherlands*[46] | MI | Myocardial infarction: Presence of clinical symptoms, elevated cardiac enzymes & ECG changes |
|  | IS | Ischaemic stroke: clinical stroke was confirmed by CT or MRI |
|  | Any arterial thrombosis | Any arterial thrombosis |
| *Mikklesen et al. (2019), Denmark* [47] | Breast cancer | Breast Cancer |
|  | Ovarian cancer | Ovarian Cancer |
|  | Endometrial cancer | Endometrial Cancer |
|  | Cervical Cancer | Cervical cancer |
|  | Bladder cancer | Bladder cancer |
|  | Renal cancer | Renal cancer |
|  | Lung cancer | Lung cancer |
|  | GI cancer | GI cancer |
|  | Brain cancer | Brain cancer |
|  | Haematological cancer | Haematological cancer |
|  | Melanoma | Melanoma |
|  | All cancers mortality | All cancers mortality |
| *Pariente et al. (2014), Israel*[16] | CNP | Cardiac non-invasive diagnostic procedures: Hospitalisation records based on ICD-9 |
|  | CIP | Cardiac invasive diagnostic procedures: Hospitalisation records based on ICD-9 |
|  | SCE | Simple cardiovascular events: Hospitalisation records based on ICD-9 |
|  | CCE | Complex cardiovascular events: Hospitalisation records based on ICD-9 |
|  | TCH | Total cardiovascular hospitalizations |
|  | Renal morbidity | Any renal outcome causing hospitalizations based on ICD-9 |
| *Parker et al. (2014) USA* [23] | MI | Myocardial infarction (Based on hospital & death records) |
|  | IS | Ischaemic stroke (Based on hospital & death records) |
| *Peters et al. (2017), China* [24] | CHD | Coronary heart disease: ICD-10 (I20–I25) |
|  | Stroke | Stroke: ICD-10, codes: (I60–I61, I63–I64) |
|  | Circulatory Disease | Circulatory disease: ICD-10, codes (I00-I99) |
| *Peters et al. (2020), China* [25] | T2DM | Type 2 Diabetes: ICD-10, codes: E10-E14 |
| *Rådestad et al. (1996), Sweden* [26] | Anxiety | Anxiety: Trait Anxiety inventory |
| *Ranthe et al. (2013), Denmark* [27] | MI | Myocardial infarction: ICD-8 code 410 and ICD-10 codes I21–I22 |
|  | IS | Cerebral infarction: (ICD-8 code 436 and ICD-10 codes I63–I64) |
|  | RHTN | Renovascular hypertension: ICD-8 code 400.39 and ICD-10 codes I12–I13 and I15.0 |
| *Rao, Ganesh, Nesai (1994), India* [59] | Breast cancer | Breast cancer: Record linkage & histological confirmation |
| *Vance et al. (1991), Australia* [30] | Anxiety | Anxiety: Two subscales from the Foulds and Bedford delusions-symptoms-states inventory (DSSI) --> reported as those with high levels of anxiety |
|  | Depression | Depression: Two subscales from the Foulds and Bedford delusions-symptoms-states inventory (DSSI) --> reported as those with high levels of depression: patient interview |
| *Winkelstein et al. (1958), USA* [51] | MI | Myocardial infarction: Characteristic history of myocardial infarction, positive ECG findings, and had to have survived for the interview |
| *Winkelstein et al. (1964), USA* [52] | IHD | Atherosclerotic heart disease: Hospital record retrieval based on ICD 420.0, 420.1, 420.2 |
| *Xu et al. (2004), China* [53] | Endometrial Cancer | Endometrial cancer: Record linkage from histologically confirmed cancer |

*CCE; Complex Cardiovascular Events, CHD; Coronary Heart Disease, CIP; Cardiac Invasive Diagnostic Procedures, CKD; Chronic Kidney Disease, CNP; Cardiac Non-invasive Diagnostic Procedures, GI; Gastrointestinal, HC; Hypercholesterolaemia, HS; Haemorrhagic Stroke, HTN; Hypertension, ICD; International Classification of Diseases, IHD; Ischaemic Heart Disease, IS; Ischaemic Stroke, MI; Myocardial Infarction, RHTN; Renovascular Hypertension, SCE; Simple Cardiovascular Events, T2DM; Type 2 Diabetes Mellitus, TCH; Total cardiovascular hospitalizations*

Table C: Covariate/Confounding variables the researchers adjusted for in the studies of miscarriage.

| ***Lead author and publication date (Country)*** | **Covariates** |
| --- | --- |
| *Adami et al. (1990), Norway* [33] |  |
| *Andalib et al. (2006), Iran* [1] |  |
| *Auger et al (2021), Canada* [2] |  |
| *Bergant et al. (1997), Austria* [4] |  |
| *Bertuccio et al. (2007), Italy* [36] |  |
| *Braem et al. (2012), Netherlands* [7] |  |
| *Brewster et al. (2005), UK* [34] | Age at bilateral oophorectomy, initial matching variables, parity, age of delivery of first child |
| *Brinton et al. (1983), USA* [35] |  |
| *Calle et al. (1995), USA* [37] | History of breast cancer, mental and occupational and dietary exposures, age |
| *Charach et al. (2018), Israel* [9] | Smoking, parity, diabetes mellitus, history of recurrent pregnancy loss |
| *Coleman et al. (2013), USA* [10] |  |
| *Cooper et al. (1999), USA* [11] |  |
| *Dick et al. (2009), Israel* [38] | Education, state of residence, BMI, family history |
| *Egerup et al (2020), Denmark* [39] |  |
| *Erlandsson et al. (2003), Sweden* [40] | Smoking, use of contraceptives, height, pre-pregnancy weight, number of births, age at first birth |
| *Gallagher et al. (2011), USA* [12] |  |
| *Goldacre et al. (2001), UK* [41] | Social class |
| *Horn et al. (a) (2019), Norway* [13] | BMI, smoking, diet, alcohol intake before pregnancy |
| *Horn et al. (b) (2019), Norway* [13] |  |
| *Janssen et al. (2006), Netherlands* [15] |  |
| *Kessous et al. (2014), Israel* [16] |  |
| *Kharazmi et al. (2011), Germany* [19] | Age, number of previous pregnancies, SBP and DBP, fasting blood glucose, total LDL, smoking, BMI, waist hip ratio, physical activity and education. |
| *Kharazmi et al. (2012), Germany* [18] |  |
| *Kharazmi, Fallah, Luoto (2010), Germany* [17] |  |
| *Kolte et al. (2015), Denmark* [54] |  |
| *La Vecchia et al. (a) (1987), Italy* [42] |  |
| *La Vecchia et al. (b), (1987), Italy* [43] |  |
| *Laing et al. (1993), USA* [44] |  |
| *Lambalk et al. (2016), Netherlands* [20] |  |
| *Lipworth et al. (1995), USA* [45] |  |
| *Maino et al. (2016), Netherlands* [46] |  |
| *Mikkelsen et al. (2019), Denmark* [47] |  |
| *Okoth et al (2022), UK* [21] |  |
| *Parazzini et al. (1992), Italy* [48] |  |
| *Pell, Smith, Walsh (2003), UK* [56] |  |
| *Peters et al. (2017), UK* [24] |  |
| *Peters et al. (2020), UK* [25] |  |
| *Ranthe et al. (2013), Denmark* [27] | History of stillbirth, history of miscarriage, years since latest miscarriage, current age, calendar period of miscarriage |
| *Reeves et al. (2006), UK* [49] | Socioeconomic and reproductive history |
| *Rosenberg et al. (1988), USA* [50] |  |
| *Schwarzman et al. (2020), Israel* [28] |  |
| *Smith et al. (2003) UK* [56] |  |
| *Toffol, Koponen, Partonen (a) (2013), Finland* [55] |  |
| *Toffol et al. (b) (2013), Finland* [55] | Marital status, number of live births, lifetime infertility and reproductive status |
| *Wagner et al. (2015), Netherlands* [31] | Gravidity, parity, primary versus secondary miscarriage, smoking, socioeconomic deprivation |
| *Winkelstein et al. (1958), USA* [51] |  |
| *Winkelstein, Rekate (1964), USA* [52] |  |
| *Xu et al. (2004), China* [53] |  |
| *Yamada et al, (2017), Japan* [32] | Age, number of deliveries, education, self-reported BMI, hours of exercise, hours of walking, smoking status, drinking status |

Table D: Covariate/Confounding variables the researchers adjusted for in the studies of stillbirth.

| *Lead author and publication date (Country)* | Covariates |
| --- | --- |
| *Auger et al (2021), Canada* [2] | Maternal age, gravidity, pre-existing comorbidity, socioeconomic status, rurality, and time period, socioeconomic disadvantage |
| *Barret et al. (2020), Sweden* [3] | Maternal age, year of delivery, country of origin, parity, body mass index, smoking, gestational diabetes, preeclampsia and small for gestational diabetes deliveries |
| *Bourne et al. (1968), UK* [5] | Subsequent pregnancies, change of doctor, number of previous pregnancies |
| *Boyle et a.l (1996), Australia* [6] | Family income, education, religious affiliation, frequency of church attendance |
| *Brinton, Hoover, Fraumeni, (1983), USA* [35] | Race, same 5-year age group |
| *Calderon-Margalit et al. (2007), Israel* [8] | Maternal age at first birth, social class according to father's occupation, ethnic origin, births that took place before the cohort. Heart disease, diabetes and other medical conditions were analysed separately and grouped together as dichotomous variable |
| *Calle et al. (1995), USA* [37] | History of breast cancer, mental and occupational and dietary exposures, age |
| *Coleman et al. (2013), Denmark* [35] | Year of birth, number of pregnancies, age at last pregnancy |
| *Egerup et al (2020), Denmark* [39] |  |
| *Gallagher et al. (2011), USA* [12] |  |
| *Gravesteen et al. (2012), Norway* [57] | Demographic and health related variables |
| *Hogue et al. (2015), USA* [58] | Known early pregnancy, maternal demographic factors, sociodemographic, behaviours and medical risks |
| *Horn et al. (2019), Norway* [13] | Age, race, height, weight, strenuous physical activity at ages 18-22, FH of HTN or diabetes, smoking status, menstrual regularity from ages 18-22, history of infertility, alcohol intake, current weight, smoking status, healthcare utilisation |
| *Hvidtjørn et al. (2016), Denmark* [14] | Sociodemographic, maternal diseases at pregnancy, placental abruption and pre-eclampsia |
| *Kharazmi et al. (2011), Germany* [19] | Education, smoking status, leisure time physical activity, alcohol, DM, hyperlipidaemia, HTN, height weight, waist hip ratio, BMI, waist circumferences, hip circumference |
| *Kharazmi et al. (2012), Germany* [18] | Education, smoking status, leisure time physical activity, alcohol, DM, hyperlipidaemia, HTN, height weight, waist hip ratio, BMI, waist circumferences, hip circumference |
| *Maino et al. (2016), Netherlands*[46] |  |
| *Mikklesen et al. (2019), Denmark* [47] | Mean age, parity, age under 30 for first live birth, previous induced abortion, diabetes mellitus, asthma, IBD, hormonal contraception, education, BMI, smoking |
| *Pariente et al. (2014), Israel*[16] |  |
| *Parker et al. (2014) USA* [23] | Variables examined: alcohol consumption, smoking, family history or early coronary heart disease, depression, metabolic equivalence |
| *Peters et al. (2017), China* [24] |  |
| *Peters et al. (2019), China* [25] | Demographic socioeconomic lifestyle factors |
| *Rådestad et al. (1996), Sweden* [26] |  |
| *Ranthe et al. (2013), Denmark* [27] | Number of live births, attained age, calendar period |
| *Rao, Ganesh, Nesai (1994), India* [59] |  |
| *Vance et al. (1991), Australia* [30] |  |
| *Winkelstein et al. (1958), USA* [51] |  |
| *Winkelstein et al. (1964), USA* [52] |  |
| *Xu et al. (2004), China* [53] |  |

References

1. Andalib A, Rezaie A, Oreizy F, Shafiei K, Baluchi S. "A study on Stress, Depression and NK Cytotoxic Potential in Women with Recurrent Spontaneous Abortion ". Iran J Allergy, Asthma Immunol. 2006;5: 9–16. Available: https://ijaai.tums.ac.ir/index.php/ijaai/article/view/126

2. Auger N, Ghadirian M, Low N, Healy-Profitós J, Wei S. Premature mortality after pregnancy loss: Trends at 1, 5, 10 years, and beyond. Obs Gynecol Reprod Biol. 2021;267: 155–160.

3. Barrett PM, McCarthy FP, Evans M, Kublickas M, Perry IJ, Stenvinkel P, et al. Stillbirth is associated with increased risk of long-term maternal renal disease: a nationwide cohort study. Am J Obstet Gynecol. 2020;223: 427.e1-427.e14. doi:10.1016/j.ajog.2020.02.031

4. Bergant A, Reinstadler K, Moncayo H, Sölder E, Heim K, Ulmer H, et al. Spontaneous abortion and psychosomatics. A prospective study on the impact of psychological factors as a cause for recurrent spontaneous abortion. Hum Reprod. 1997;12: 1106–1110. doi:10.1093/HUMREP/12.5.1106

5. Bourne S. The psychological effects of stillbirths on women and their doctors. J R Coll Gen Pract. 1968;16: 103–112. Available: https://www.ncbi.nlm.nih.gov/pmc/articles/PMC2236635/

6. Boyle FM, Vance JC, Najman JM, Thearle MJ. The mental health impact of stillbirth, neonatal death or sids: Prevalence and patterns of distress among mothers. Soc Sci Med. 1996;43: 1273–1282. doi:10.1016/0277-9536(96)00039-1

7. Braem M, Onland-Moret N, Schouten L, Kruitwagen R, Lukanova A, Allen N, et al. Multiple miscarriages are associated with the risk of ovarian cancer: results from the European Prospective Investigation into Cancer and Nutrition. PLoS One. 2012;7. doi:10.1371/JOURNAL.PONE.0037141

8. Calderon-Margalit R, Friedlander Y, Yanetz R, Deutsch L, Manor O, Harlap S, et al. Late stillbirths and long-term mortality of mothers. Obstet Gynecol. 2007;109: 1301–1308. doi:10.1097/01.AOG.0000264548.10767.ea

9. Charach R, Sheiner E, Beharier O, Sergienko R, Kessous R. Recurrent pregnancy loss and future risk of female malignancies. Arch Gynecol Obstet 2018 2984. 2018;298: 781–787. doi:10.1007/S00404-018-4868-4

10. Coleman PK, Reardon DC, Calhoun BC. Reproductive history patterns and long-term mortality rates: A Danish, population-based record linkage study. Eur J Public Health. 2013;23: 569–574. doi:10.1093/eurpub/cks107

11. Cooper G, Ephross S, Weinberg C, Baird D, Whealan E, Sandler D. Menstrual and Reproductive Risk Factors for Ischemic Heart Disease. Epidemiology. 1999;1: 9.

12. Gallagher LG, Davis LB, Ray RM, Psaty BM, Gao DL, Checkoway H, et al. Reproductive history and mortality from cardiovascular disease among women textile workers in Shanghai, China. Int J Epidemiol. 2011;40: 1510–1518. doi:10.1093/ije/dyr134

13. Horn J, Tanz LJ, Stuart JJ, Markovitz AR, Skurnik G, Rimm EB, et al. Early or late pregnancy loss and development of clinical cardiovascular disease risk factors: a prospective cohort study. BJOG An Int J Obstet Gynaecol. 2019;126: 33–42. doi:10.1111/1471-0528.15452

14. Hvidtjørn D, Wu C, Schendel D, Thorlund Parner E, Brink Henriksen T. Mortality in mothers after perinatal loss: A population-based follow-up study. BJOG An Int J Obstet Gynaecol. 2016;123: 393–398. doi:10.1111/1471-0528.13268

15. Janssen HJEM, Cuisinier MCJ, Hoogduin KAL, De Graauw KPHM. Controlled prospective study on the mental health of women following pregnancy loss. https://doi.org/101176/ajp1532226. 2006;153: 226–230. doi:10.1176/AJP.153.2.226

16. Kessous R, Shoham-Vardi I, Pariente G, Sergienko R, Holcberg G, Sheiner E. Recurrent pregnancy loss: a risk factor for long-term maternal atherosclerotic morbidity? Am J Obstet Gynecol. 2014;211: 414.e1-414.e11. doi:10.1016/J.AJOG.2014.05.050

17. Kharazmi E, Fallah M, Luoto Ri. Miscarriage and risk of cardiovascular disease. Acta Obstet Gynecol Scand. 2010;89: 284–288. doi:10.3109/00016340903380758

18. Kharazmi E, Lukanova A, Teucher B, Groß ML, Kaaks R. Does pregnancy or pregnancy loss increase later maternal risk of diabetes? Eur J Epidemiol. 2012;27: 357–366. doi:10.1007/s10654-012-9683-9

19. Kharazmi E, Dossus L, Rohrmann S, Kaaks R. Pregnancy loss and risk of cardiovascular disease: A prospective population-based cohort study (EPIC-Heidelberg). Heart. 2011;97: 49–54. doi:10.1136/hrt.2010.202226

20. Lambalk C, Kolman E, Van Den Belt-Dusebout A, Spaan M, Krul I, Groeneveld E, et al. Recurrent pregnancy loss is not associated with an increased breast cancer risk. Abstr 32nd Annu Meet ESHRE. 2016; 31.

21. Okoth K, Subramanian A, Chandan J, Adderley N, Thomas, GN Nirantharakumar, K Antza C. Long term miscarriage-related hypertension and diabetes mellitus. Evidence from a United Kingdom population-based cohort study. PLoS One. 2022;17.

22. Pariente G, Shoham-Vardi I, Kessous R, Sergienko R, Sheiner E. Is stillbirth associated with long-term atherosclerotic morbidity? American Journal of Obstetrics and Gynecology. Mosby Inc.; 2014. pp. 416.e1-416.e12. doi:10.1016/j.ajog.2014.07.032

23. Parker DR, Lu B, Sands-Lincoln M, Kroenke CH, Lee CC, O’Sullivan M, et al. Risk of cardiovascular disease among postmenopausal women with prior pregnancy loss: The women’s health initiative. Ann Fam Med. 2014;12: 302–309. doi:10.1370/afm.1668

24. Peters SAE, Yang L, Guo Y, Chen Y, Bian Z, Tian X, et al. Pregnancy, pregnancy loss, and the risk of cardiovascular disease in Chinese women: Findings from the China Kadoorie Biobank. BMC Med. 2017;15. doi:10.1186/s12916-017-0912-7

25. Peters SAE, Yang L, Guo Y, Chen Y, Bian Z, Sun H, et al. Pregnancy, pregnancy loss and the risk of diabetes in Chinese women: findings from the China Kadoorie Biobank. Eur J Epidemiol. 2020;35: 295–303. doi:10.1007/s10654-019-00582-7

26. Radestad I, Steineck G, Sjogren B, Nordin C. Psychological complications after stillbirth—influence of memories and immediate management: Population based study. BMJ. 1996;312: 1505–1508. doi:10.1136/bmj.312.7045.1505

27. Ranthe MF, Andersen EAW, Wohlfahrt J, Bundgaard H, Melbye M, Boyd HA. Pregnancy loss and later risk of Atherosclerotic disease. Circulation. 2013;127: 1775–1782. doi:10.1161/CIRCULATIONAHA.112.000285

28. Schwarzman P, Paz Levy D, Walfisch A, Sergienko R, Bernstein E, Sheiner E. Maternal history of recurrent pregnancy loss and long-term risk of thromboembolic events. J Reprod Immunol. 2020;138. doi:10.1016/J.JRI.2020.103084

29. Smith GCS, Pell JP, Walsh D. Spontaneous loss of early pregnancy and risk of ischaemic heart disease in later life: retrospective cohort study. BMJ. 2003;326: 423–424. doi:10.1136/BMJ.326.7386.423

30. Vance JC, Foster WJ, Najman JM, Embelton G, Thearle MJ, Hodgen FM. Early parental responses to sudden infant death, stillbirth or neonatal death. Med J Aust. 1991;155: 292–297. doi:10.5694/j.1326-5377.1991.tb142283.x

31. Wagner MM, Bhattacharya S, Visser J, Hannaford PC, Bloemenkamp KW. Association between miscarriage and cardiovascular disease in a Scottish cohort. Heart. 2015;101: 1954–1960. doi:10.1136/HEARTJNL-2015-307563

32. Yamada K, Iso H, Cui R, Tamakoshi A. Recurrent Pregnancy Loss and Cardiovascular Disease Mortality in Japanese Women: A Population-Based, Prospective Cohort Study. J Stroke Cerebrovasc Dis. 2017;26: 1047–1054. doi:10.1016/J.JSTROKECEREBROVASDIS.2016.12.018

33. Adami H, Bergström R, Lund E, Meirik O. Absence of association between reproductive variables and the risk of breast cancer in young women in Sweden and Norway. Br J Cancer. 1990;62: 122–126. doi:10.1038/BJC.1990.242

34. Brewster D, Stockton D, Dobbie R, Bull D, Beral V. Risk of breast cancer after miscarriage or induced abortion: a Scottish record linkage case-control study. J Epidemiol Community Health. 2005;59: 283–287. doi:10.1136/JECH.2004.026393

35. Brinton LA, Hoover R, Fraumeni JF. Reproductive factors in the aetiology of breast cancer. Br J Cancer. 1983;47: 757–762. doi:10.1038/bjc.1983.128

36. Bertuccio P, Tavani A, Gallus S, Negri E, La Vecchia C. Menstrual and reproductive factors and risk of non-fatal acute myocardial infarction in Italy. Eur J Obstet Gynecol Reprod Biol. 2007;134: 67–72. doi:10.1016/J.EJOGRB.2007.01.005

37. Calle EE, Mervis CA, Wingo PA, Thun MJ, Rodriguez C, Heath CW. Spontaneous abortion and risk of fatal breast cancer in a prospective cohort of United States women. Cancer Causes Control. 1995;6: 460–468. doi:10.1007/BF00052187

38. Dick M-LB, Siskind V, Purdie DM, Green AC. Incomplete pregnancy and risk of ovarian cancer: results from two Australian case–control studies and systematic review. Cancer Causes Control 2009 209. 2009;20: 1571–1585. doi:10.1007/S10552-009-9402-3

39. Egerup P, Mikkelsen A, Kolte A, Westergaard, D Rasmussen S, Knop F, Lidegaard Ø, et al. Pregnancy loss is associated with type 2 diabetes: a nationwide case-control study. Diabetologia. 2020;63: 1521–1529.

40. Erlandsson G, Montgomery S, Cnattingius S, Ekbom A. Abortions and breast cancer: record-based case-control study. Int J cancer. 2003;103: 676–679. doi:10.1002/IJC.10877

41. Goldacre MJ, Kurina LM, Seagroatt V, Yeates D. Abortion and breast cancer: a case-control record linkage study. J Epidemiol Community Heal. 2001;55: 336–337. doi:10.1136/JECH.55.5.336

42. Vecchia C La, Decarli A, Parazzini F, Gentile A, Negri E, Cecchetti G, et al. General epidemiology of breast cancer in Northern Italy. Int J Epidemiol. 1987;16: 347–355. doi:10.1093/ije/16.3.347

43. Vecchia C La, Decarli A, Franceschi S, Gentile A, Negri E, Parazzini F. Menstrual and reproductive factors and the risk of myocardial infarction in women under fifty-five years of age. Am J Obstet Gynecol. 1987;157: 1108–1112. doi:10.1016/S0002-9378(87)80271-5

44. Laing AE, Demenais FM, Williams R, Kissling G, Chen VW, Bonney GE. Breast cancer risk factors in African-American women: the Howard University Tumor Registry experience. J Natl Med Assoc. 1993;85: 931–939. Available: /pmc/articles/PMC2568204/?report=abstract

45. Lipworth L, Katsouyanni K, Ekbom A, Michels KB, Trichopoulos D. Abortion and the risk of breast cancer: A case-control study in greece. Int J Cancer. 1995;61: 181–184. doi:10.1002/IJC.2910610207

46. Maino A, Siegerink B, Algra A, Martinelli I, Peyvandi F, Rosendaal FR. Pregnancy loss and risk of ischaemic stroke and myocardial infarction. Br J Haematol. 2016;174: 302–309. doi:10.1111/bjh.14043

47. Mikkelsen AP, Egerup P, Ebert JFM, Kolte AM, Nielsen HS, Lidegaard Ø. Pregnancy Loss and Cancer Risk: A Nationwide Observational Study. EClinicalMedicine. 2019;15: 80–88. doi:10.1016/j.eclinm.2019.08.017

48. Parazzini F, Vecchia C La, Negri E, Franceschi S, Bocciolone L. Menstrual and reproductive factors and breast cancer in women with family history of the disease. Int J Cancer. 1992;51: 677–681. doi:10.1002/IJC.2910510502

49. Reeves GK, Kan SW, Key T, Tjønneland A, Olsen A, Overvad K, et al. Breast cancer risk in relation to abortion: Results from the EPIC study. Int J Cancer. 2006;119: 1741–1745. doi:10.1002/ijc.22001

50. Rosenberg L, Palmer JR, Kaufman DW, Strom BL, Schottenfeld D, Shapiro S. Breast cancer in relation to the occurrence and time of induced and spontaneous abortion. Am J Epidemiol. 1988;127: 981–989. doi:10.1093/oxfordjournals.aje.a114901

51. Winkelstein W, Stenchever MA, Lilienfeld AM. Occurrence of pregnancy, abortion, and artificial menopause among women with coronary artery disease: A preliminary study. J Chronic Dis. 1958;7: 273–286. doi:10.1016/0021-9681(58)90085-7

52. Winkelstein W, Rerate AC. Age trend of mortality from coronary artery disease in women and observations on the reproductive patterns of those affected. Am Heart J. 1964;67: 481–488. doi:10.1016/0002-8703(64)90094-8

53. Xu WH, Xiang YB, Ruan ZX, Zheng W, Cheng JR, Dai Q, et al. Menstrual and reproductive factors and endometrial cancer risk: Results from a population-based case-control study in urban shanghai. Int J Cancer. 2004;108: 613–619. doi:10.1002/ijc.11598

54. Kolte AM, Olsen LR, Mikkelsen EM, Christiansen OB, Nielsen HS. Depression and emotional stress is highly prevalent among women with recurrent pregnancy loss. Hum Reprod. 2015;30: 777–782. doi:10.1093/HUMREP/DEV014

55. Toffol E, Koponen P, Partonen T. Miscarriage and mental health: Results of two population-based studies. Psychiatry Res. 2013;205: 151–158. doi:10.1016/J.PSYCHRES.2012.08.029

56. Smith GCS, Pell JP, Walsh D. Spontaneous loss of early pregnancy and risk of ischaemic heart disease in later life: retrospective cohort study. BMJ Br Med J. 2003;326: 423. doi:10.1136/BMJ.326.7386.423

57. Gravensteen IK, Helgadottir LB, Jacobsen EM, Sandset PM, Ekeberg Ø. Long-term impact of intrauterine fetal death on quality of life and depression: A case-control study. BMC Pregnancy Childbirth. 2012;12. doi:10.1186/1471-2393-12-43

58. Hogue CJR, Parker CB, Willinger M, Temple JR, Bann CM, Silver RM, et al. The association of stillbirth with depressive symptoms 6-36 months post-delivery. Paediatr Perinat Epidemiol. 2015;29: 131–143. doi:10.1111/ppe.12176

59. Rao DN, Ganesh B, Desai PB. Role of reproductive factors in breast cancer in a low-risk area: A case—control study. Br J Cancer. 1994;70: 129–132. doi:10.1038/bjc.1994.261
