# Supplementary figures and images for: Fetal loss and long-term maternal morbidity and mortality: A systematic review and meta-analysis
Source: PLoS Med. 2024 Feb 9;21(2):e1004342. doi: 10.1371/journal.pmed.1004342 (PMC10857720; doi:10.1371/journal.pmed.1004342)

Supplementary Information: Abstract and Manuscript PRISMA Checklist


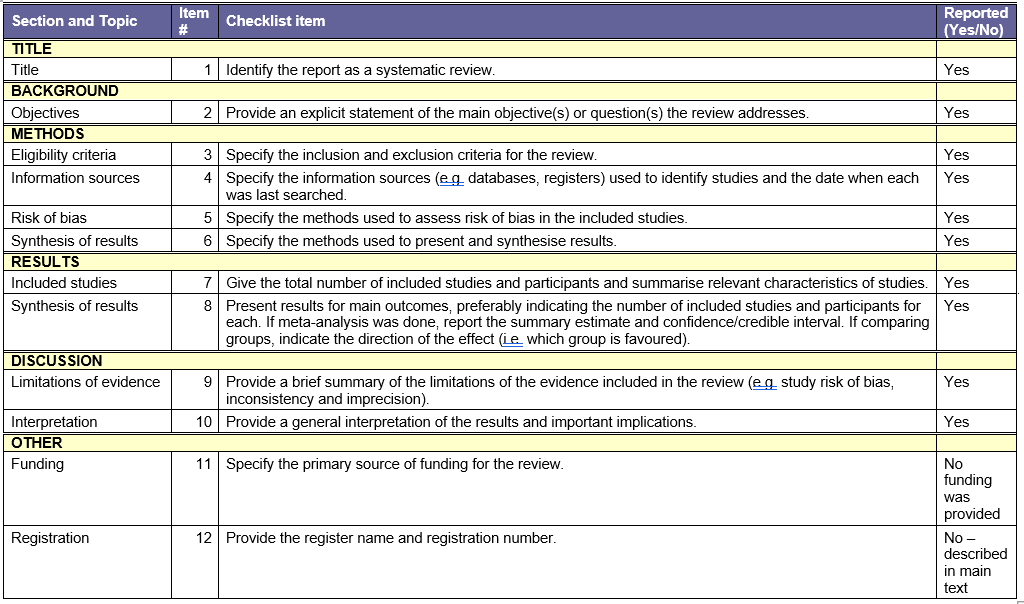


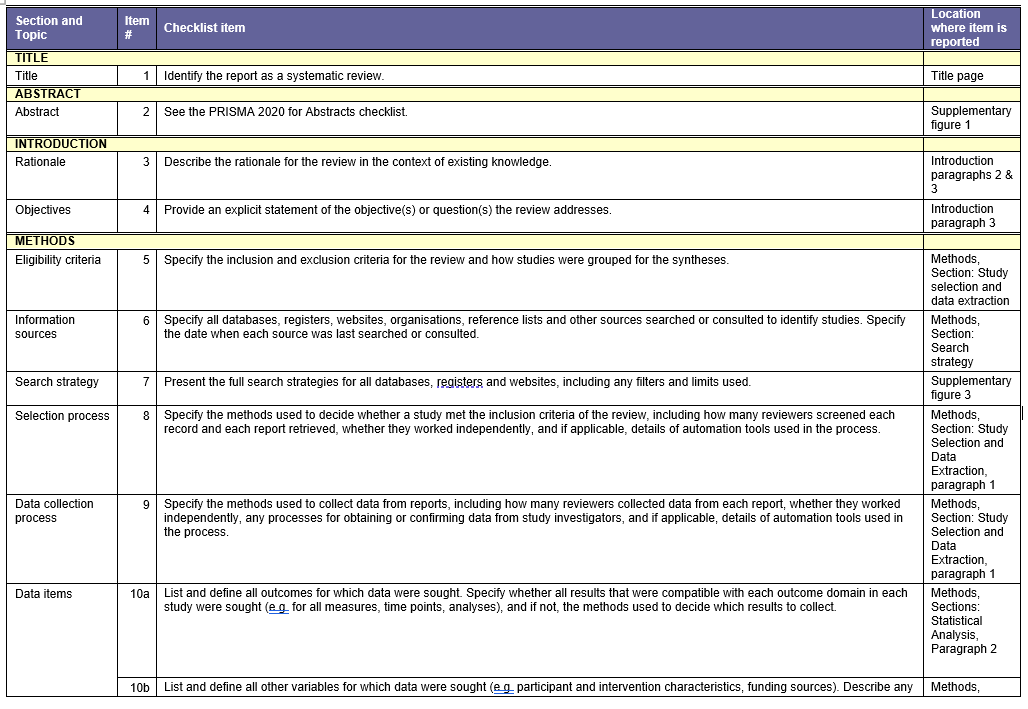


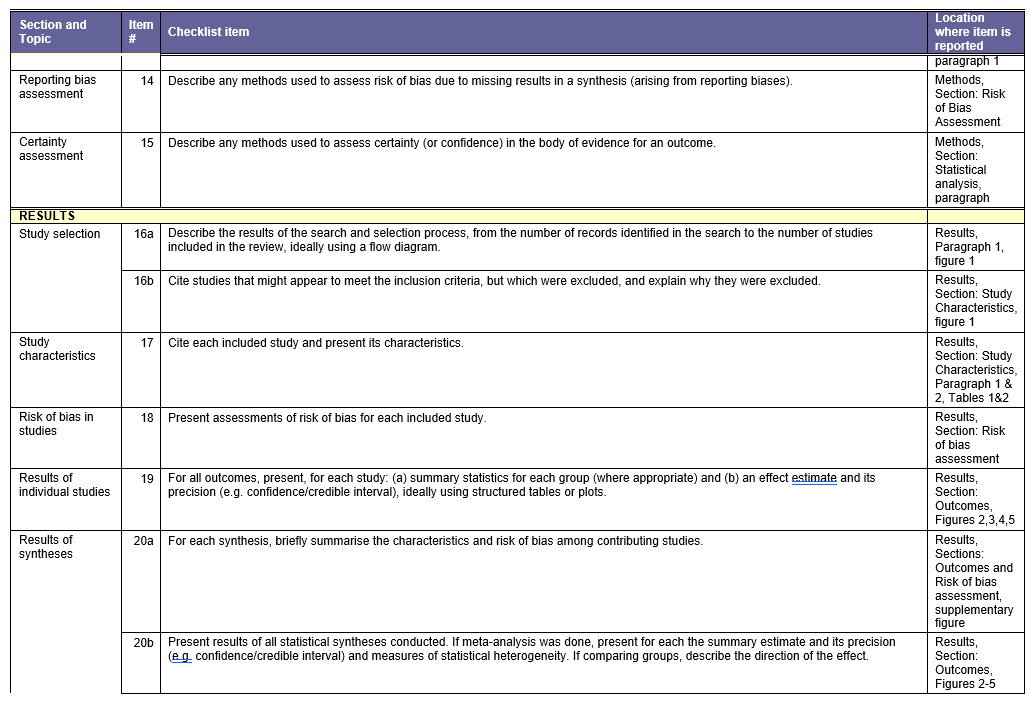


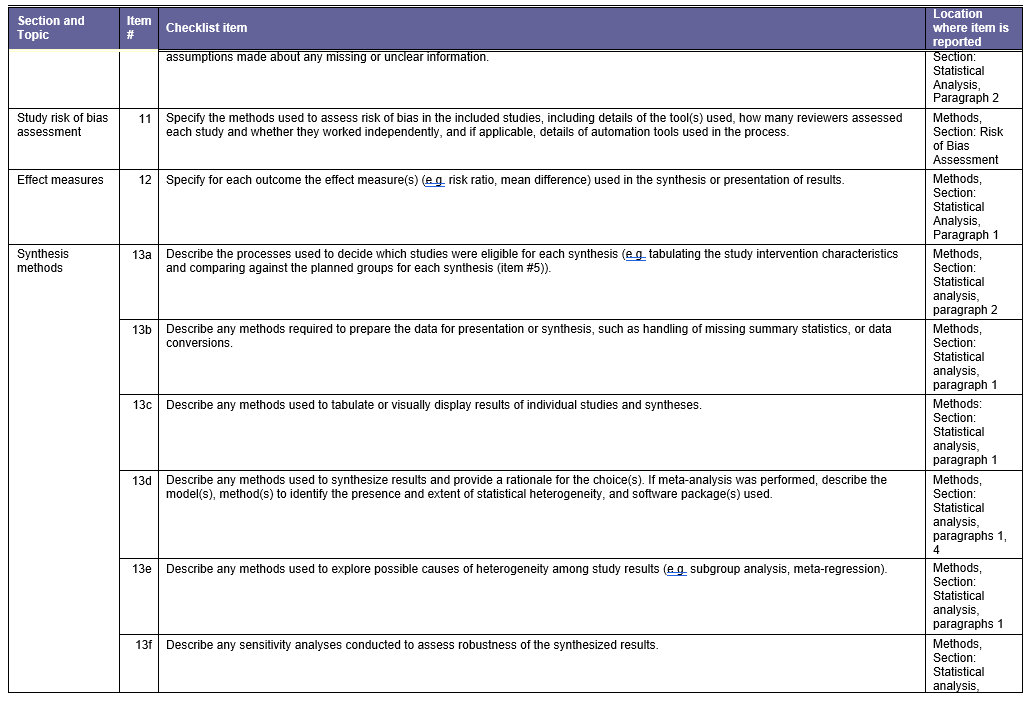

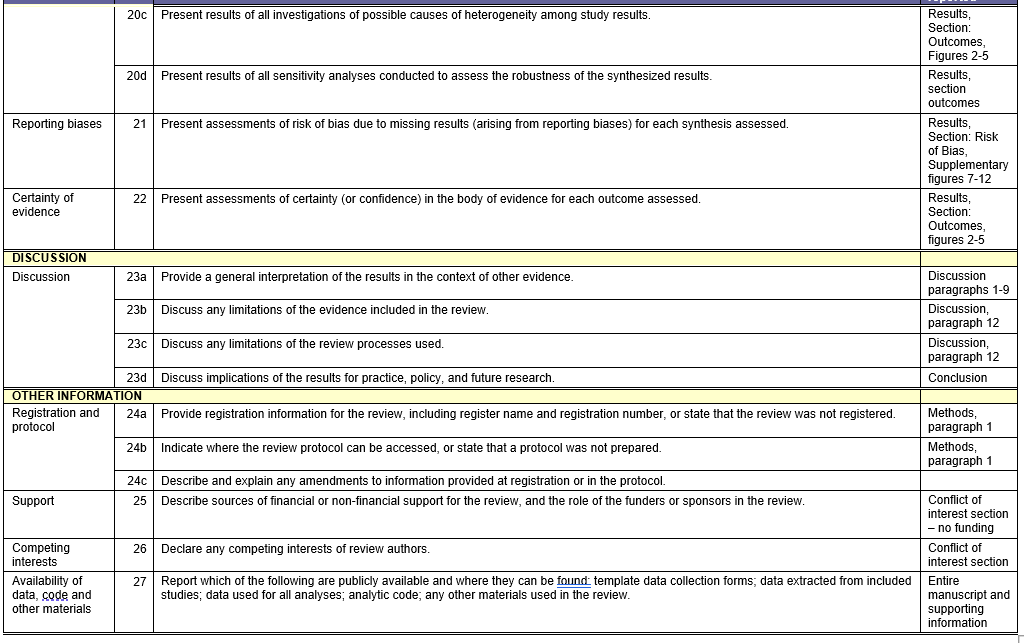

Supplement: S1 PRISMA Checklist — (DOCX) [file pmed.1004342.s002.docx]
