# Supplementary material for: Fetal loss and long-term maternal morbidity and mortality: A systematic review and meta-analysis
Source: PLoS Med. 2024 Feb 9;21(2):e1004342. doi: 10.1371/journal.pmed.1004342 (PMC10857720; doi:10.1371/journal.pmed.1004342)
Supplement: Attachment — Submitted filename: vlachou.pdf [file pmed.1004342.s003.pdf]

This paper presents a systematic review with meta–analysis of studies of foetal loss and various maternal outcomes.

I was asked for a statistical report and I interpret that to include all aspects of the design and conduct of the study.

1 Points of detail

Page 5 Just for the record there is now an updated PRISMA guideline (Page et al., 2021a,b). I suspect any differences are fairly marginal.

Page 5 'The following [. . .] were searched . . . '. The actual database names seem to have gone missing although they are listed in the abstract. Page 4 tells us that the majority of the cases of stillbirth are in low– and middle–income countries. The existing search revealed few studies in LMICs. Various empirical studies have indicated that the main bibliographic databases have varied and incomplete coverage of material not in English (Pilkington et al., 2005; Shenderovich et al., 2016), and have limited coverage of material from low and middle–income country journals (Kieling et al., 2009; Syed Sheriff et al., 2008). A wider search, for instance in some of the WHO–sponsored ones, might throw up more studies.

Page 5 and 6 The inclusion criteria (page 5) tell us that women with previous experience of loss were excluded but page 6 tells us it was a stratification variable. Does page 5 mean previous history of the outcomes not the types of loss? This needs clarifying.

Page 7 I am not sure that using an arbitrary cut–off for heterogeneity is helpful. As Rücker et al. (2008) have argued it is quite misleading when primary studies are large and give precise estimates. Many of the studies included here had more than a quarter of a million women enrolled which is large by most people's standards. The discussion on page 18 could also benefit from recognising this issue.

Page 9 Perhaps summarise the numbers per country to save the reader having to go through Table 1 and tot them up. The geographical spread is helpful to assess generalisability.

Page 9 Mean age at loss 23, mean age at follow up 69, mean follow up period 16 years. 69 - 23 = 46. I can see the figures need not agree exactly but that is a rather large discrepancy.

Page 11 Not presenting a summary estimate is fine but we should still see

the primary studies and a bit more detail about just why they differ so much, the current text is rather cryptic.

Page 12 The sentence starting 'Moreover, endometrial' seems to appear twice.

Page 15 I applaud the caution of stating first to our knowledge but the rest of the discussion occasionally reverts to saying the first.

Page 18 Finding few studies from LMIC seems to me worth mentioning as a limitation here.

Tables 1 and 2 Would these be easier to read if they were in landscape so that more width could be given to some of the columns thus avoiding having studies split over more than one page?

Forest plots The standard RevMan wording 'favours stillbirth' seems to me rather unfortunate as having a higher risk of disease is hardly favourable.

2 Points of more substance

2.1 Some comments on the evidence base

It seems to me that there may be more going on in the datasets than is currently presented. To illustrate this I took the first result mentioned in the abstract and did some reanalysis on it. I modelled log risk ratios using inverse variance weighting. I estimated τ^2 using REML and used the Knapp–Hartung adjustment (Knapp and Hartung, 2003). This has been shown to give improved confidence interval coverage (Viechtbauer, 2005). I used the R metafor package in version 3.8-1.

Rates in the control arm

The most striking feature is that the baseline rates vary in the non–stillbirth arm. The largest rate is more than 300 times larger than the smallest. Even if we ignore the two oldest studies on the basis that the world has moved on since 1958 the largest is still more than 100 times the smallest. Having a wide range of situations is obviously a good thing from the point of view of generalisability but there must come a point when it becomes doubtful that the same processes are at work in high incidence settings and low incidence settings.

Prediction interval

Riley et al. (2011) have suggested that interpretation of random effects models should also incldue a prediction interval. I calculate that a 95% interval is from 0.82 to 3.76 on the risk ratio scale. In contrast to the confidence interval which tells us about the precision with which we have estimated the mean of the distribution of effects the prediction interval tells us where the next study is likely to lie. In this case the prediction interval includes the null value.

Confidence interval for heterogeneity

I estimate the 95% confidence interval for τ^2 to stretch from 0.02 to 0.69 which suggests that our estimates here must be regarded as rather uncertain. This obviously affects a random effects model by introducing more uncertainty.

Would risk difference be more appropriate?

There are arguments both for and against using risk difference rather than risk ratio. If we re–analyse using risk difference we find a central estimate of 0.019 with 95% confidence interval from -0.002 to 0.040 which obviousl (just) includes the null value.

What conclusions would I draw?

My feeling from this re–analysis is that the conclusion should be that the evidence base is not yet very strong. I have not examined any of the others. None of this of course is the authors' fault, we can only meta–analyse what we can find, not what we would have liked to find.

2.2 Evidence of absence

In various places the authors seem to suggest that not being able to demonstrate an effect means that there is no effect. This is not the case and those instances would be better re–written as some form of 'We were unable to demonstrate an effect.

3 Summary

This is a report of a substantial piece of work. The length of my comments should not be interpreted as a value judgment on it but as a justification of my caution in accepting that the case is in any way closed.

Michael Dewey

References

C Kieling, H Herrman, V Patel, and J J Mari. Indexation of psychiatric journals from low– and middle–income countries: a survey and a case study. *World Psychiatry*, 8:40–44, 2009.

G Knapp and J Hartung. Improved tests for a random effects meta–regression with a single covariate. *Statistics in Medicine*, 22:2693–2710, 2003.

M J Page, J E McKenzie, P M Bossuyt, I Boutron, T C Hoffmann, C D Mulrow, L Shamseer, J M Tetzlaff, E A Akl, S E Brennan, R Chou, J Glanville, J M Grimshaw, A Hróbjartsson, M M Lalu, T Li, E W Loder, E Mayo-Wilson, S McDonald, L A McGuiness, L A Stewart, J Thomas, A C Tricco, V A Welch, P Whiting, and D Moher. The PRISMA 2020 statement: an updated guideline for reporting systematic reviews. *British Medical Journal*, 372:n71, 2021a.

M J Page, D Moher, P M Bossuyt, I Boutron, T C Hoffmann, C D Mulrow, L Shamseer, J M Tetzlaff, E A Akl, S E Brennan, R Chou, J Glanville, J M Grimshaw, A Hróbjartsson, M M Lalu, T Li, E W Loder, E Mayo-Wilson, S McDonald, L A McGuiness, L A Stewart, J Thomas, A C Tricco, V A Welch, P Whiting, and J E McKenzie. PRISMA 2020 explanation and elaboration: updated guidance and exemplars for reporting systematic reviews. *British Medical Journal*, 372:n160, 2021b.

K Pilkington, A Boshnakova, M Clarke, and J Richardson. 'No language restrictions' in database searches: what does this really mean? *The Journal of Alternative and Complementary Medicine*, 11:205–207, 2005.

R D Riley, J P T Higgins, and J J Deeks. Interpretation of random effects meta–analyses. *British Medical Journal*, 342:964–967, 2011.

G Rücker, G Schwarzer, J R Carpenter, and M Schumacher. Undue reliance on I^2 in assessing heterogeneity may mislead. *BMC Medical Research Methodology*, 8(79), 2008.

Y Shenderovich, M Eisner, C Mikton, F Gardner, J Liu, and J Murray. Methods for conducting systematic reviews of risk factors in low– and middle–income countries. *BMC Medical Research Methodology*, 16(32), 2016.

R J Syed Sheriff, C E Adams, P Tharyan, M Jayaram, L Duley, and the PRACTIHC Mental Health Group. Randomised trials relevant to mental

health conducted in low and middle–income countries: a survey. *BMC Psychiatry*, 8(69), 2008.

W Viechtbauer. Bias and efficiency of meta–analytic variance estimators in the random–effects model. *Journal of Educational and Behavioral Statistics*, 30:261–293, 2005.
