# Supplementary material for: Fetal loss and long-term maternal morbidity and mortality: A systematic review and meta-analysis
Source: PLoS Med. 2024 Feb 9;21(2):e1004342. doi: 10.1371/journal.pmed.1004342 (PMC10857720; doi:10.1371/journal.pmed.1004342)
Supplement: Attachment — Submitted filename: Response to reviewers_2023 August.docx [file pmed.1004342.s004.docx]

**ACADEMIC EDITOR COMMENTS**

| Comments | Response |
| --- | --- |
| The reviewers raise a number of issues with this paper - the majority of which I agree with.  The potential for confounding is very large and this was not addressed. Even if papers did appropriately adjust for potential confounding (which was not presented) the potential for residual confounding is high. It is well recognised that pregnancy complications and cardiovascular disease share similar risk factors. | We thank the academic editor for his comment. We agree that the potential for confounding may be substantial; to mitigate this potential source of bias, we revised our methodology to combine the most adjusted ratios in our analysis as recommended by the statistician and as seen in a recent systematic review and meta-analysis published by Kyriacou et al (2022). We re-analysed our data for both the miscarriage and stillbirth outcomes using the inverse variance weight random-effects analysis combining the most adjusted odds ratios (ORs), risk ratios (RRs) and hazard ratios (HRs) to produce pooled RR and 95% confidence intervals under the rare outcome assumption. Whenever this was not possible based on the information provided in the primary studies, the original dichotomous weighted random effect Mantel-Haenszel statistical analysis with the raw data was used. Our original unadjusted analysis was included in the supplementary material. This change is reflected in the methods section on page 9 lines 152-158 which now reads: “Meta-analysis was conducted in situations where at least 3 studies reported the same exposure-outcome combination. Data synthesis and analysis was performed using inverse variance weighted random-effects meta-analysis to combine the most adjusted reported odds ratios (ORs), RRs and hazard ratios (HRs) to produce pooled RRs and 95% confidence intervals (CIs) under the rare outcome assumption. Additionally, we repeated the analysis using the unadjusted raw data from each studies as dichotomous outcomes with the Mantel-Haenszel statistical method using a random effect model (supplementary material).” Additionally, our results presented in the abstract, results section and figures have been changed to the new values obtained by our revised analysis. However, we do recognise that the pooled results can never be fully adjusted for all confounding factors, as it is conditional to the adjustment available in the primary studies. We have included this limitation in our discussion on page 33 lines 423-426 which reads: “Also, we note that while we used the most adjusted results from the studies, we recognise that there is some degree of confounding which cannot be accounted for. This is because the included studies are observational and thus, we extracted aggregated data with variable degrees of adjustment.” |
| The authors argue that even if the association is due to shared risk factors it is still useful to identify a high risk population so they can be followed up. I disagree. There are much stronger cardiovascular risk factors than stillbirth and well validated risk tools for assessment of cardiovascular risk, therefore the clinical benefit of using stillbirth to identify risk is questionable (especially when the evidence for the association is not strong). | While we agree there are established risk factors for cardiovascular disease (such as changes in blood pressure, cholesterol levels etc) these are often identified in an older population, where traditional screening tools such as annual reviews of blood pressure are carried out. Our main conclusion is that other risk factors such as reproductive exposures may be present much earlier in the life course. Most of women experiencing pregnancy losses many of whom are young are unlikely to have developed established cardiovascular risk factors. As a consequence, early identification of women who are likely to go on to develop cardiovascular events, when preventative measure and lifestyle modifications are more efficient, would be missed. In addition, these women are likely to have numerous encounters with healthcare professionals for their reproductive challenges but less likely to see a specialist for cardiovascular risk prevention, therefore, it would be a missed opportunity not to identify high risk women earlier before other risk factors appear. |
| The timing of exposure (pregnancy loss) and outcome (maternal health) is crucial in these analyses- ie did the pregnancy loss precede development of illness or did the pregnancy loss occur on the background of illness. The intervals between exposure and outcomes also needs to be considered. These were not addressed. | Thank you for your comment. One of our exclusion criteria when screening studies was the condition that women did not have any previous diagnosis of the condition of interest. Studies in which pregnancy loss did not precede illness were excluded from our systematic review and meta-analysis. The intervals between exposure and outcomes were noted for each of the studies and are presented in tables 1&2. Our protocol specified that outcomes which were diagnosed / observed less than 1 year from pregnancy loss were excluded. The mean exposure time in each of the arms (miscarriage and stillbirth) are described in the results on page 11 lines 183-184 which reads: “The mean follow-up period for outcome assessment from index pregnancy for the miscarriage cohort was 19 while for stillbirth this was 17 years.” |
| There was a lack of detail in the definitions of stillbirth and miscarriage. The lack of detail on gestation of loss, definitions, and cause or suspected cause of loss is problematic as different causes are likely to have different associations with maternal disease. The authors state “ Stillbirth and miscarriage are pregnancy losses occurring at different gestational ages and it has been hypothesised that they may share an underlying aetiology related to placental malformation, thrombotic and vascular changes.”.  This may be true for proportion of, mainly later miscarriages, and some stillbirths. However, early miscarriages are more likely to have other causes (for example chromosomal abnormality) and numerically these are by far the most common type of loss. | We thank the academic editor for this comment. We do recognise that the definitions used were not mentioned in the original submission. This is because there is variation in these definitions across countries and studies. For example, in the USA and Canada miscarriage is defined as loss before 20 weeks of gestation whereas in the UK it is described as loss before 24 weeks. Additionally, some of the included studies did not describe the gestational cut-off used to distinguish between miscarriage and stillbirth. We classified the exposure (miscarriage or stillbirth) based on the definitions used by each study and we have now presented the definitions used in each study in tables 1 and 2.  We have now amended the methods section in our manuscript. Page 9 lines: 150-151 which now reads “Given the variability in gestational age definitions for pregnancy loss across different countries, the pregnancy loss exposure (miscarriage or stillbirth) was defined based on the definition provided by each study.” In addition to this, we amended the study characteristics section in results, to present the reported gestational age in the studies we included. Page 11, lines: 185-188 which now reads “Most of the studies did not provide a gestation cut off for the definition of stillbirth or miscarriage (37/59). The remaining studies provided definitions varying between 20- and 28-weeks gestational age as the cut off in distinguishing between stillbirth and miscarriage, with only 3 specifically reporting first trimester losses.”  While we agree with what the editor has stated, first trimester losses are commonly associated with factors such as chromosomal abnormalities, in practice in the vast majority of cases no such abnormalities are found in the cytogenetics, and in fact the vast proportion of recurrent losses in particular have no known cause identified. There anecdotal evidence that thrombotic and vascular changes contribute to losses, thereby, women history of with recurrent miscarriages or stillbirth are often advised to take low dose aspirin in a subsequent pregnancy. |
| Absolute rates/risk reduction and numbers were not included. This makes interpretation difficult. | We would like to thank academic editor for highlighting this. We have now added the raw values in the figures and in the Results section in the form of percentage as seen in pages: 23-, lines: 216, 220, 232-233, 240, 249, 258-259, 267, 270, 282-283, 288, 298-299, 304, 308-309, 313-314, 326, 329. |
| As the statistical reviewers additional analyses showed - there is enormous variation in rate of IHD in non-stillbirth arm. This may relate to follow up time or other differences - but it does raise questions on the rationale for combining these data. The other additional analyses by the reviewer suggest that the evidence base is not strong. | We want to thank the academic editor for highlighting this. We acknowledge that in meta-analysis we are limited to the data provided in the primary studies. We have taken various steps to mitigate some of the concerns raised including:   1. By estimating ratios rather prevalence, the results are more likely to be representative of the magnitude of the existing associations and as rightly pointed more generalisable. 2. We have revised our analysis and conducted an inverse variance analysis where SE have been estimated and shown in the forest plots, so risk differences can be calculated. However, we feel the ratios are more clinically meaningful and easier to be explained to patients/ lay audience.   We agree with the academic editor that the evidence may not be strong enough because of the degree of uncertainty, however, we still feel that we cannot ignore the results suggesting that reproductive exposures such as a history of fetal loss can facilitate earlier identification of women at higher risk of future disease. We have edited our conclusions in the discussion to show more explicitly this. |

**EDITORS’ COMMENTS**

| Comments | Response |
| --- | --- |
| GENERAL  Please respond to all editor and reviewer comments detailed below in full. | We have responded to all comments point by point below. |
| Please cite the reference numbers in square brackets (e.g., “We used the techniques developed by our colleagues [19] to analyze the data”). Citations should be preceding punctuation. | Thank you for highlighting. The referencing style was changed throughout the manuscript as per PLOS protocol, ie: Vancouver referencing style and using square brackets. |
| Please cite your Supporting Information as outlined here:  <https://eur01.safelinks.protection.outlook.com/?url=https%3A%2F%2Fjournals.plos.org%2Fplosmedicine%2Fs%2Fsupporting-information&data=05%7C01%7C%7C4cff425a5e47426b4f7208db56ecfd06%7C569df091b01340e386eebd9cb9e25814%7C0%7C0%7C638199348392039121%7CUnknown%7CTWFpbGZsb3d8eyJWIjoiMC4wLjAwMDAiLCJQIjoiV2luMzIiLCJBTiI6Ik1haWwiLCJXVCI6Mn0%3D%7C3000%7C%7C%7C&sdata=wxyGhFIqBa3bF3GkRzzQhYYom7JRFWXHEYLmkTe%2Bg6U%3D&reserved=0> | Our supporting information has been uploaded as per PLOS guidelines provided by the editors. |
| Please include page numbers and line numbers in the manuscript file. Use continuous line numbers (do not restart the numbering on each page). | Thank you for noting. Page and line numbers were added throughout the manuscript |
| Please ensure to be consistent in the use of numbers as words or numerals. | All numbers have been changed to numerals throughout the manuscript. |
| Please adjust the number format using a consistent format (1000 or 1,000). | Number format has been changed to the format with the comma throughout manuscript for consistency. |
| Please check your manuscript carefully for grammar, spelling and punctuation. | The manuscript has been reviewed and we have hopefully addressed all issues with grammar, spelling and punctuation. |
| ABSTRACT  Please report your abstract according to PRISMA for abstracts, following the PLOS Medicine abstract structure (Background, Methods and Findings, Conclusions)  <https://eur01.safelinks.protection.outlook.com/?url=http%3A%2F%2Fwww.plosmedicine.org%2Farticle%2Finfo%3Adoi%2F10.1371%2Fjournal.pmed.1001419&data=05%7C01%7C%7C4cff425a5e47426b4f7208db56ecfd06%7C569df091b01340e386eebd9cb9e25814%7C0%7C0%7C638199348392039121%7CUnknown%7CTWFpbGZsb3d8eyJWIjoiMC4wLjAwMDAiLCJQIjoiV2luMzIiLCJBTiI6Ik1haWwiLCJXVCI6Mn0%3D%7C3000%7C%7C%7C&sdata=WKqOC9h0OLTKNFe4aZ1FxHVOa1xT0O9n01TlD2FY470%3D&reserved=0> . | The abstract has been amended to reflect PLOS Journal guidelines as specified by the editors and is seen on pages 2-3, lines: 20-64 |
| Please provide the types of study designs included, and synthesis/appraisal methods. | Thank you for your comment. The types of studies included are mentioned in the “Study Selection and Data Extraction” section of the methods on page 8 lines 129-131 and it reads: “Case reports, letters, in vitro and animal studies were also excluded, and cohort, case-control and cross-sectional studies were included”. Additionally, synthesis / appraisal methods have been described in the “Statistical analysis” section of the methods on page 9 lines 153-158 and it reads: “Data synthesis and analysis was performed using inverse variance weighted random-effects meta-analysis to combine the most adjusted reported odds ratios (ORs), RRs and hazard ratios (HRs) to produce pooled RRs and 95% confidence intervals (CIs) under the rare outcome assumption. Additionally, we repeated the analysis using the unadjusted raw data from each study as dichotomous outcomes with Mantel-Haenszel statistical method using a random effect model.” |
| Abstract Methods and Findings:  * Please ensure that all numbers presented in the abstract are present and identical to numbers presented in the main manuscript text.  PLOS Medicine requests that main results are quantified with 95% CIs as well as p values. Please include. When reporting p values please report as p<0.001 and where higher as the exact p value p=0.002, for example. For the purposes of transparent data reporting, if not including the aforementioned please clearly state the reasons why not. | Thank you for noting. The manuscript and Abstract have been checked to ensure there is no discrepancies between the reported numbers.  Thank you for your comment and apologies for the omission. All reported results have now included 95% CI and p-values throughout the abstract and manuscript as seen in page 3, lines 44-48, pages 23-28, lines: 217, 222-224, 234, 241, 249-250, 262, 268, 271, 283, 288-289, 300, 306, 310-311, 318, 327-328, 330 |
| Please include any important dependent variables that are adjusted for in the analyses. | Following the recommendations of both the academic editor and reviewers, we have revised our analysis to include the most adjusted ratios (odds ratios, risk ratios and hazard ratios) from each study. Each study has adjusted for different variables and since this is not an individual participant data meta-analysis, we were unable to control for the same variables in all analyses. This is reflected in the methods section page 8 lines 146-152 and discussed as a limitation of the study page 33 lines 423-428. We have included the variables used for adjustment in each primary study in supplementary tables 3 and 4 for the reader’s reference. We accept that this is a limitation that applies to every aggregated data meta-analysis. |
| Please note the use of commas to separate upper and lower bounds, as opposed to hyphens as these can be confused with reporting of negative values.  * In the last sentence of the Abstract Methods and Findings section, please describe the main limitation(s) of the study's methodology.  Please remove the “Key words” paragraph from the Abstract. | Thank you for your comment. Hyphens have been replaced throughout the manuscript.  The “Key Words’ paragraph was removed. |
| AUTHOR SUMMARY  At this stage, we ask that you include a short, non-technical Author Summary of your research to make findings accessible to a wide audience that includes both scientists and non-scientists. The Author Summary should immediately follow the Abstract in your revised manuscript. This text is subject to editorial change and should be distinct from the scientific abstract. Please see our author guidelines for more information:  <https://eur01.safelinks.protection.outlook.com/?url=https%3A%2F%2Fjournals.plos.org%2Fplosmedicine%2Fs%2Frevising-your-manuscript%23loc-author-summary&data=05%7C01%7C%7C4cff425a5e47426b4f7208db56ecfd06%7C569df091b01340e386eebd9cb9e25814%7C0%7C0%7C638199348392039121%7CUnknown%7CTWFpbGZsb3d8eyJWIjoiMC4wLjAwMDAiLCJQIjoiV2luMzIiLCJBTiI6Ik1haWwiLCJXVCI6Mn0%3D%7C3000%7C%7C%7C&sdata=8oEPulwoUDS6IC%2FvMf3VMnk0P82Z2zTRf3QPXa8%2BYE8%3D&reserved=0>.  The summary should include 2-3 single sentence, individual bullet points under each of the questions.  It may be helpful to review currently published articles for examples which can be found on our website here  <https://eur01.safelinks.protection.outlook.com/?url=https%3A%2F%2Fjournals.plos.org%2Fplosmedicine%2F&data=05%7C01%7C%7C4cff425a5e47426b4f7208db56ecfd06%7C569df091b01340e386eebd9cb9e25814%7C0%7C0%7C638199348392039121%7CUnknown%7CTWFpbGZsb3d8eyJWIjoiMC4wLjAwMDAiLCJQIjoiV2luMzIiLCJBTiI6Ik1haWwiLCJXVCI6Mn0%3D%7C3000%7C%7C%7C&sdata=VouSeildtYRJNXaUa%2F3xl5cLOTlmeoDm0A5G6KhhpCI%3D&reserved=0> | An author summary has been added in page 4 lines 66-73 and reads:  “This manuscript summarises the long-term development of maternal disease following pregnancy loss in currently published literature. Our work suggests that history of stillbirth is associated with an increased risk of heart disease, stroke, type 2 diabetes mellitus, and kidney disease by 56%, 71%, 86%, 17%, and 97% respectively, while with a lower risk of breast cancer by 20%. We were unable to show an association between history of miscarriage and incidence of long-term disease. History of stillbirth can be used as an early marker for identification of women at high risk of future disease that may benefit from screening and lifestyle modifications. Prospective studies of good methodology are needed to further replicate our findings, especially the risk reduction in breast cancer.” |
| INTRODUCTION  p.4: Please change “[…] at risk of developing of future cardiovascular, […]” to “[…] at risk of developing future cardiovascular, […]”. | Thank you for you recommendation. The above has been changed as recommended. |
| METHODS AND RESULTS  Please report your SR/MA according to the PRISMA guidelines provided at the EQUATOR site.  <https://eur01.safelinks.protection.outlook.com/?url=http%3A%2F%2Fwww.equator-network.org%2Freporting-guidelines%2Fprisma%2F&data=05%7C01%7C%7C4cff425a5e47426b4f7208db56ecfd06%7C569df091b01340e386eebd9cb9e25814%7C0%7C0%7C638199348392039121%7CUnknown%7CTWFpbGZsb3d8eyJWIjoiMC4wLjAwMDAiLCJQIjoiV2luMzIiLCJBTiI6Ik1haWwiLCJXVCI6Mn0%3D%7C3000%7C%7C%7C&sdata=GTxx%2Bn98VPs6mA1PgOx%2BUHBcbnXr4VXibA8o538oF3Y%3D&reserved=0> | Our meta-analysis has been reported according to the PRISMA guidelines and this is reflected in figure 2 in the supplementary information. |
| Please provide the completed PRISMA checklist.  When completing the checklist, please use section and paragraph numbers, rather than page numbers. | The PRISMA checklist has been added as figure 2 of the supplementary information. |
| Please add the following statement, or similar, to the Methods: "This study is reported as per the Preferred Reporting Items for Systematic Reviews and Meta-Analyses (PRISMA) guideline (S1 Checklist)."Please update your PROSPERO entry (ID: CRD42021225370). | Thank you for your comment. We have now updated the reference according to the new PRISMA guidelines published in 2020, and the sentence on page 7 lines 102-104 reads: “We conducted a systematic review and meta-analysis of existing literature in accordance with the recommended methods described by Cochrane and reported using the Preferred Reporting Items for Systematic reviews and Meta-analysis (PRISMA) guidelines (S1 Checklist). [13] |
| Please update your search to the present time. | We have now updated the search in June 2023. As a consequence, all relevant sections of the manuscript have been updated. |
| Please evaluate evidence of publication bias. | Publication bias was examined using funnel plots of standard error against risk ratio for each of the outcomes. This was only performed in outcomes that included at least 10 studies as recommended in literature (Sterne et al (2011). The methods section “Risk of bias assessment” on page 8-9 line 145-147 has been amended to reflect this and it now reads: “Publication bias was assessed using funnel plots with axis of standard error against risk ratio (RR) in outcomes with more than 10 studies.”. The interpretation of the funnel plots was described in the results, section “Risk of bias” on pages 23, lines 210-211 which now reads: “Publication bias was assessed in 2 outcomes; IHD and breast cancer in the miscarriage arm. Both funnel plots appear symmetrical denoting low risk of publication bias (supplementary figure 7).”  The funnel plots have been added in the supplementary information (fig 7). |
| PLOS Medicine requests that main results are quantified with 95% CIs as well as p values. Please include. When reporting p values please report as p<0.001 and where higher as the exact p value p=0.002, for example. For the purposes of transparent data reporting, if not including the aforementioned please clearly state the reasons why not. | All results have now been amended to include 95% CIs as well as p-values throughout the manuscript. |
| Please include any important dependent variables that are adjusted for in the analyses. | Following the recommendations of both the academic editors and reviewers in this process, we revised our analysis to include the most adjusted ratios (odds ratios, risk ratios and hazard ratios) from each study. Each study has adjusted for different variables and since this is not an individual participant data meta-analysis, we were unable to control for the same variables in all analyses. This is reflected in the methods section page 9 lines 152-158 and discussed as a limitation of the study 33 lines 423-426. We have included the variables used for adjustment in each primary study in the supplementary tables 3 and 4 for the reader’s reference. We accept that this is a limitation to our study but applies to every aggregated data meta-analysis. |
| Suggest reporting statistical information as detailed above – see under ABSTRACT | We have now amended this. |
| p. 5: “The following electronic databases were searched from inception to May 2022 to identify studies exploring the effects of fetal loss on subsequent maternal morbidity and mortality” – Please include the name of the databases included in your study. | Thank you for highlighting this. The text has been amended, and page 7 lines 107-109 now reads: “Electronic databases including MEDLINE, EMBASE, AMED, BNI, CINAHL, and the Cochrane Library were searched from inception to June 2023 to identify studies exploring the association of fetal loss with subsequent maternal morbidity and mortality”. |
| p.8: Please correct “The included 32 cohort studies […]”. | Thank you for noting. The sentence on page 11 lines: 176-177 was amended and both this and the previous sentence read as: “We identified 59 studies which were included in the review. The included 32 of these were cohort studies of which 16 were retrospective cohort and 16 prospective cohorts.” |
| p.8.: Under “Study Characteristics” you write that of 25 case control studies two were retrospective cross-sectional studies. This would mean that your review included 57 studies in total. Please re-phrase the sentence if you want to describe the retrospective cross-sectional studies as a distinct set of studies. | The sentence on page 11 lines 177-178 has been rephrased and reads: “There were 25 case-controlled studies and 2 retrospective cross-sectional studies.” |
| p.9: Please define “across Europe.”. | Thank you for highlighting. The sentence has been amended and page 11 lines 1180-183 read as: “to “Most studies were conducted in high-income countries in North America (n=12) Europe (n=35), Australia (n=2) and Israel (n=6). The remaining 4 studies were conducted in Asia (Japan: n=1, China: n=1, India: n=1, Iran: n=1) including 1 high, 2 middle and 1 low-income countries.” |
| p.9: Due to repetition, please re-phrase “The mean maternal age at the time of fetal loss was on average 23 years at the time of loss (Pell et al., 2004) […]”. Editorial suggestion: “The mean maternal age was on average 23 years at the time of fetal loss (Pell et al., 2004) […]”. | The phrase has been removed as we have rechecked our data and calculations and recognised that there was a mistake (as noted by the reviewers). The discrepancy arises from the difference in maternal age reported between case-control and cohort studies, with the former reporting age at outcome presentation and the latter age at recruitment (after pregnancy loss). Given the variability in the maternal age reported we decided to exclude this information from our results section. We have recalculated these figures and we now report the average follow-up in each of the arms of the study (miscarriage: 19 years, stillbirth:17 years). Page 11 lines 183-184 now reads: “The mean follow-up period for outcome development from index pregnancy was 19 for the miscarriage cohort while was 17 years for stillbirth.” |
| p.9: Please write “Table 1”/“Figure 1” instead of “table 1”/”figure 1” and check throughout your manuscript. | The words ‘Table’ and “Figure’ changed as recommended throughout the manuscript. |
| p.9: “There were 29 studies looking at women experiencing a stillbirth with most being as low risk of bias, with only one older study identified as being at high risk of bias”. – change “as low risk” to “at low risk”. | The phrase was amended as recommended on page 23 lines 208-209. |
| p.9: Please elaborate/clarify whether the bias identified could be attributed to the study 'vintage' or refer to “only one study...” instead of “only one older study”. | The word ‘older’ has been removed from the sentence on page 23 lines 206-207. |
| p.9: Please change “[…] went on to have IHD, […]” to “[…] developed IHD, […]”. | The phrase was changed as recommended above and page 23 line 216 now reading: “[…] developed IHD […]” |
| p.10: “Similarly, recurrent loss compared with a single loss resulted in a pooled estimate 1.13 (95%  CI: 0.89-1.44, I2 = 71%)”. – change “in a pooled estimate 1.13” to “in a pooled estimate of 1.13”. | Thank you for your comment. The sentence is now amended, and page 23 line 222-224 reads: “Similarly, recurrent loss compared with a single loss resulted in a pooled estimate of 1.13 (95% CI [0.89,1.44]; p=0.32, I^2^ = 71%). |
| p.11: “The pooled risk ratio […]” – once introduced as abbreviations, please ensure consistency in the use of these (e.g., RR for risk ratio). Please check your manuscript carefully for this. | The manuscript was checked to ensure that once abbreviations were introduced, they were consistently used in the text and in the figures. |
| p.11: Under “Malignant disease”, please split the first sentence into two separate sentences starting the second one with “Data were extracted from all studies but Goldacre et al […]” and explain why the study of Goldacre et al was not included in your analysis. | We have amended the sentence on page 26 lines 285-288 based on your recommendation. The study was excluded as it only provided no extractable data for our meta-analyses. |
| p.12: “[…], while cervical malignancy risk was assessed one paper (Mikkelsen et al., 2019).” – change to “[…], while cervical malignancy risk was assessed in one paper (Mikkelsen et al., 2019).” | The phrase on page 28 line 312 has been amended and reads: “while cervical malignancy risk was assessed in 1 paper [61]” |
| p.13: Under “Malignant disease”, please check grammar and syntax of the last sentence/paragraph starting with “Three studies combined to assess [..]”. | The paragraph has been amended and page 28 lines 316-318 reads:  “3 studies assessed the risk of all cancer-related mortality in women with a previous history of stillbirth (n=8,609; events: 81) [29,35, 61]. compared to those without such loss (controls: n=885,036, events: n=6,245). Analysis yielded a RR of 1.17, (97% CI: [0.95,-1.45]; p=0.15, I^2^ = 0%) (Fig 4d).” |
| p.14: “Future risk of long term depression, anxiety, stress and alcohol dependence were assessed after pregnancy loss in five studies of women with a history of miscarriage […] and in six studies in women with a history of stillbirth [..] respectively” – please change “long term” to “long-term” and remove “respectively”. | Thank you for your comment. The sentence on page 28 lines 321-323 has been amended as suggested. Additionally, the words ‘long term’ were replaced with ‘long-term’ throughout the manuscript. |
| DISCUSSION  Please avoid assertions of primacy ("We report for the first time....").  Please present and organize the Discussion as follows: a short, clear summary of the article's findings; what the study adds to existing research and where and why the results may differ from previous research; strengths and limitations of the study; implications and next steps for research, clinical practice, and/or public policy; one-paragraph conclusion. | Thank you for this, we have now updated the manuscript, and the first sentence of the discussion on page 29 lines 336-337 now reads: “We conducted a systematic review and meta-analysis examining the association between fetal loss and long-term maternal morbidity or mortality in women experiencing either miscarriage or stillbirth”.  We have edited part of the discussion to align with these suggestions. |
| p.15: “Our results suggest that women experiencing stillbirth have an increased risk of developing ischaemic heart disease and cerebrovascular disease by 74 and 71% respectively” – please ensure to add units when necessary and check carefully throughout your manuscript. | Thank you for highlighting this, we have now updated the text on page 29 line 339 and checked the manuscript for other similar omissions. The sentence now reads: “Our results suggest that women experiencing stillbirth have an increased risk of developing ischaemic heart disease and cerebrovascular disease by 56% and 71% respectively”. The figure was changed to reflect the results from the inverse variance analysis which was adopted throughout the manuscript following recommendations of the PLOS statistician. |
| p.16: “However, other risk factors such as such as hyperlipidaemia, hyperglycaemia, […]” – please remove the duplicate “such as”. | The repeated words on page 30 lines 368-369 have now been removed. |
| FIGURES  Please provide titles and legends for all figures (including those in Supporting Information files).  For all Figures, please ensure that you have complied with our figures requirements <https://eur01.safelinks.protection.outlook.com/?url=http%3A%2F%2Fjournals.plos.org%2Fplosmedicine%2Fs%2Ffigures&data=05%7C01%7C%7C4cff425a5e47426b4f7208db56ecfd06%7C569df091b01340e386eebd9cb9e25814%7C0%7C0%7C638199348392039121%7CUnknown%7CTWFpbGZsb3d8eyJWIjoiMC4wLjAwMDAiLCJQIjoiV2luMzIiLCJBTiI6Ik1haWwiLCJXVCI6Mn0%3D%7C3000%7C%7C%7C&sdata=U8wSDs1n7nBqj4BXj8x%2BSFhzj3BdROToAt2qgxrmguE%3D&reserved=0>. | Titles and legends have been provided for all figures including those in the Supporting information files on pages 41-44.  Figures have been modified to reflect the PLOS Medicine style. |
| Please consider avoiding the use of red and green in order to make your figure more accessible to those with colour blindness. | We have now changed the figure format and present the figures and tables in grey scale. |
| Please ensure to define abbreviations used in your figures and tables. | Thank you for your comment. All abbreviations have been defined in the figures and tables. |
| REFERENCES  PLOS uses the numbered citation (citation-sequence) method and first six authors, et al. | The references have now been altered to reflect the PLOS Medicine bibliography style (page 35-41). |

**REVIEWER 1 COMMENTS**

| **Comments** | **Response** |
| --- | --- |
| Reviewer #1: The authors conducted a systematic review and meta-analysis of 56 studies on the association between pregnancy loss and long-term maternal morbidity and mortality. The authors found that women with a history of stillbirth increased risk of ischaemic heart disease, cerebrovascular disease, and renal disease compared with no stillbirth. Additionally, women with a history of stillbirth had a lower risk of breast cancer. The review is comprehensive, well-structured, and the conclusions are supported by the evidence. The article has already been revised once (I was not consulted at the initial revision). | We would like to thank the reviewer for their positive comments. |
| There seem to be 2 issues in this paper:  -Definition of exposure - stillbirth bs miscarriage are not adequately defined in the methods or elsewhere. The definition provided in the introduction is not necessarily the one that will be used by many of the studies reviewed. | We thank the reviewer for this comment. We do recognise that we have not given a universal definition. The reason for this is because there are different definitions across countries and studies. Miscarriage is defined as the spontaneous loss of pregnancy before the fetus reaches viability. The term therefore includes all pregnancy losses from the time of conception until 20 to 24 weeks of gestation. It should be noted that advances in neonatal care have resulted in more babies surviving birth before 24 weeks of gestation. For example, in the USA and Canada miscarriage is defined as loss before 20 weeks of gestation whereas in the UK it is still described as loss before 24 weeks. Additionally, some of the selected studies did not describe the gestational cut-off used to distinguish between miscarriage and stillbirth. Therefore, we defined the exposure based on the categorization provided by each paper. The gestational cut-off used by individual studies is now presented in tables 1 and 2 (where this information was available to us). We have now amended the methods section on page 9 lines 150-151 to reflect the above point and the section now reads: “Given the variability in gestational age definitions for pregnancy loss across different studies, we used the definition provided by each study.” In addition to this, we amended the study characteristics section in results on page 11 lines 185-188, to present the reported gestational age in the studies we included. The aforementioned section now reads “Most of the studies did not provide a definition of stillbirth or miscarriage (37/59). The remaining studies provided definitions varying between 20- and 28-weeks gestational age in defining miscarriage and stillbirth, with only 3 specifically reporting first trimester losses.” |
| -Potential for confounding (stillbirth may be secondary to underlying morbidity). | Many thanks for your comment. We do agree that there is potential for confounding and as such we have amended our methodology and analysis based on your recommendations, that of the statistician and based on the published methodology of a similar study by Kyriacou et al (2022). We re-analysed our data in both the miscarriage and stillbirth arms using the inverse variance weight random-effects analysis combining the most adjusted odds ratios (ORs), RRs and hazard ratios (HRs) to produce pooled RR and 95% confidence intervals under the rare outcome assumption. Whenever the most adjusted ratio was not available in the primary studies, the raw data with dichotomous weighted random effect Mantel-Haenszel analysis was used. Our original unadjusted analysis was included in the supplementary material. This change is reflected in the methodology section, lines the section page 9 lines 152-158 which now reads: “Meta-analysis was conducted in situations where at least 3 studies reported the same exposure-outcome combination. Data synthesis and analysis was performed using inverse variance weighted random-effects meta-analysis to combine the most adjusted reported odds ratios (ORs), RRs and hazard ratios (HRs) to produce pooled RRs and 95% confidence intervals (Cis) under the rare outcome assumption. Additionally, we repeated the analysis using the unadjusted raw data from each study as dichotomous outcomes with the Mantel-Haenszel statistical method using a random effect model.”  Additionally, the results presented in the abstract, results section and figures have been updated. However, we do recognise that this analysis can never be fully adjusted for all confounding factors, especially considering that the majority of our studies are observational involving aggregated data that each may have adjusted for different confounders. Thus, we included a relevant entry in our discussion as a limitation on page 33 lines: 423-426 which reads: “while we used the most adjusted results from the studies, we recognise that there is some degree of confounding which cannot be accounted for. This is because the included studies are observational and thus we extracted aggregated data with variable degrees of adjustment.” |
| Introduction  -the authors give the definition of stillbirth from the WHO. However, most countries in this review will likely be from high income regions where the definition of stillbirth differs. In Canada, we use 20 weeks as the cutoff, same for the US. | We want to thank the reviewer for the comment. We agree that is since most studies originate from high income countries including USA, Canada, and Northern Europe it is possible to include descriptions of the definitions of pregnancy loss used in the index studies. We have updated the introduction to reflect this on page 5 lines 79-86. “There is a variation in the definitions used for pregnancy loss. WHO defines a loss occurring before 28 weeks of gestation as a miscarriage, while a still birth is a loss after 28 weeks in accordance to the ICD-10 criteria. [1]. These definitions are not used universally, and miscarriage is a fetal loss before the fetus reaches viability and still birth is a fetal loss beyond this gestation. The advances in neonatal and complex pregnancy care in addition to health system variation have resulted in different cut-off in gestation. For example, in the United States of America (USA) and Canada miscarriage is defined as a pregnancy loss before 20 weeks gestation [2] [3], while in the United Kingdom (UK) a gestational age of 24 weeks is usually used, however, this may change in line with the recent British Association of Perinatal Medicine (BAPM) recommendations that resuscitation can be considered between 22 to 24 weeks [4] [5].” |
| Methods:  * Search Strategy (page 5): Provide a detailed search strategy that is transparent and repeatable, including the names of the databases searched, key words, search terms (both free-text and controlled vocabulary terms). Additionally, as the search was up to May 2022. This seems far back and could be updated to a more recent end date. | We do appreciate the need to include the full keyword strategy, databases used and search terms. This was previously omitted for the because of word count, however we have now added this back in Methods in the Search Strategy section on page 7 lines 107-115 which now reads: “Electronic databases including MEDLINE, EMBASE, AMED, BNI, CINAHL, and the Cochrane Library were searched from inception to June 2023 to identify studies exploring the association of fetal loss with subsequent maternal morbidity and mortality. We used the terms “foetal loss”, “miscarriage”, “foetal death”, “stillbirth” and “spontaneous abortion” including alternative spellings for exposure. Our outcome keyworks included “cardiovascular disease”, “myocardial infarction”, “hypertension”, “cerebrovascular disease”, “stroke”, “type 2 diabetes”, “cancer”, “malignancy”, “anxiety”, “depression”, “mortality” and “morbidity” with relevant synonyms, alternative spellings and MESH terms. There were no language or date restrictions applied to the searches and we only included human studies. (See S3 Fig for detailed strategy)”  Additionally, a new search was performed, and it is now updated to June 2023. This is now reflected in the PRISMA flow chart (figure 1 and methods page 6 line 102) abstract, and results sections. |
| * Study selection (page 5): Consider providing the inclusion criteria. | Thank you for highlighting this point. We have included the inclusion criteria in the section Study selection and data extraction, pages 8 lines 123-131. The paragraph now reads: “Studies were included if they examined long-term incidence of cardiovascular, metabolic, malignant, mental health or any other morbidity in a population of women who had experienced fetal loss without a previous history of these conditions. Long-term was defined as any morbidity developing at least a year after pregnancy loss. Additionally, we included studies investigating long-term all-cause mortality after pregnancy loss. Studies reporting maternal morbidity following a pregnancy termination or outcomes as a direct complication of the pregnancy were excluded from our review. Case reports, letters, in vitro and animal studies were also excluded, and cohort, case-control and cross-sectional studies were included.” In addition to this, the inclusion criteria are also outlined in the PROSPERO protocol cited in the methods section. |
| * Data extraction (page 6): As the included studies were observational, it should be possible to extract the confounding factors for each study as well as the adjusted hazards ratios or risk ratios. | We want to thank the reviewer for highlighting this point. We have now included this information in supplementary tables 3 and 4 and we have revised the analysis so we present the pooled ratios of the most adjusted ratios from each primary study. |
| * Exposure (page 6): Miscarriage or stillbirth should be clearly defined, including gestational age cut-off. | We thank the reviewer for this comment. We do recognise that we have not given a universal definition. The reason for this is because there are different definitions across countries and studies. Miscarriage is defined as the spontaneous loss of pregnancy before the fetus reaches viability. The term therefore includes all pregnancy losses from the time of conception until 20 to 24 weeks of gestation. It should be noted that advances in neonatal care have resulted in more babies surviving birth before 24 weeks of gestation. For example, in the USA and Canada miscarriage is defined as loss before 20 weeks of gestation whereas in the UK it is still described as loss before 24 weeks. Additionally, some of the selected studies did not describe the gestational cut-off used to distinguish between miscarriage and stillbirth. Therefore, we defined the exposure based on the categorization provided by each paper. The gestational cut-off used by individual studies is now presented in tables 1 and 2 (where this information was available to us). We have now amended the methods section on page 9 lines 150-151 to reflect the above point and the section now reads: “Given the variability in gestational age definitions for pregnancy loss across different studies, we used the definition provided by each study.” In addition to this, we amended the study characteristics section in results on page 11 lines 185-188, to present the reported gestational age in the studies we included. The aforementioned section now reads “Most of the studies did not provide a definition of stillbirth or miscarriage (37/59). The remaining studies provided definitions varying between 20- and 28-weeks gestational age in defining miscarriage and stillbirth, with only 3 specifically reporting first trimester losses.” |
| * Outcomes (page 6): The outcomes could be better justified and organized. For example, "ischaemic heart disease (IHD), cerebrovascular disease, overall circulatory disease" - how about other cardiovascular diseases such as heart failure? What diseases were included in overall circulatory disease? | We acknowledge that we were limited by using the outcomes individual studies used in their primary analyses. Where study investigators state specific cardiovascular outcomes such as ischaemic heart disease, we have analysed them separately. In instances where cardiovascular or overall circulatory disease is reported, we were limited to include these outcomes without further subcategorization. The included studies reported cardiovascular outcomes as defined by the international categorization of disease (ICD) (version 9 or 10 depending on the publication date) which encompass acute rheumatic fever, chronic rheumatic heart disease, hypertensive disease, ischaemic heart disease, diseases of the pulmonary circulation, cardiovascular and cerebrovascular disease and other associated diseases We have included the outcomes extracted from each study in supplementary tables 1 and 2. |
| * Statistical analysis (page 6): Describe the statistical analysis method used for calculating a summary measure of effect size in a meta-analysis, such as the Mantel Haenszel, Peto, or other method.  * As the included studies were observational, the authors could pool the adjusted hazards ratios or adjusted risk ratios adjusted for confounding factors, rather than crude risk ratios only. This would help as stillbirth may simply be a marker of underlying morbidity which is the cause of future diseases. | We used a dichotomous analysis with the Mantel-Haenszel statistical method and random effects model for aggregating unadjusted results and we then carried out a second, more adjusted analysis based on recommendations from the academic editor and reviewers. This involved using inverse variance analysis to combine the most adjusted RRs, ORs and HRs under the rare outcome hypothesis wherever this was possible. The text in the methodology section page has been amended as described in our response in the comment below.  We appreciate that this is a valid point and we thank the reviewer for highlighting this. This was also noted by the academic editor and the other reviewers and thus we revised our methodology to include the most adjusted results in our analysis. We re-analysed our data in both the stillbirth and miscarriage arms using the inverse variance weight random-effects analysis combining the most adjusted RRs and hazard HRs to produce pooled RR and 95% confidence intervals under the rare outcome assumption. Page 9 lines 152-158 now reads: “Meta-analysis was conducted in situations where at least 3 studies reported the same exposure-outcome combination. Data synthesis and analysis was performed using inverse variance weighted random-effects meta-analysis to combine the most adjusted reported odds ratios (ORs), RRs and hazard ratios (HRs) to produce pooled RRs and 95% confidence intervals (CIs) under the rare outcome assumption. Additionally, we repeated the analysis using the unadjusted raw data from each studies as dichotomous outcomes with the Mantel-Haenszel statistical method using a random effect model.” Additionally, our results presented in the abstract, results section and figures have been changed to the new values obtained by our revised analysis. However, we do recognise that the pooled results can never be fully adjusted for all the potential confounding factors, as it is conditional to the adjustment available in the primary studies. We have included this limitation in our discussion on page 33 lines: 423-426 which reads: “Also, we note that while we used the most adjusted results from the studies, we recognise that there is some degree of confounding which cannot be accounted for. This is because the included studies are observational and thus, we extracted aggregated data with variable degrees of adjustment.” |
| Results  -Is there a way to make the text more legible -page 8 is very hard to read. | We have condensed the text as far as we can to improve readability and added subheadings where possible. |
| -hard to interpret the findings without the definition of the exposure | We thank the reviewer for expressing their concern and we have now tried to address this limitation. Metanalyses are limited to the definitions provided by primary studies, however, the majority of the studies are from high income countries where miscarriage is defined as fetal loss before viability and this can vary from 20 to 24 weeks, which reflects the advances in neonatal care. Of the included studies, 37/59 studies did not provide any gestational cutoff to distinguish between stillbirth and miscarriage, and from the remaining studies, stillbirth was defined as fetal loss after 20 weeks of gestation. The exception to this were 3 studies reporting first trimester losses miscarriages. Only one of these was included in our meta-analysis for the risk of breast cancer after miscarriage. Exclusion of this study did not change the results meaningfully. We have included the different definitions used in each study in supplementary tables 1 and 2 for reference. We have discussed the small variation in the gestational age as a limitation in our discussion. However, we feel that this does not undermine the findings of our study which is a pragmatic approach which reflects clinical practice and highlights that despite the small variation in definitions between countries, we can still use obstetric history and specifically history of fetal losses to identify earlier women at risk of overt disease later in life. |
| Discussion  -Lots of potential for confounding due to unmeasured characteristics of women, especially comorbidity such as preeclampsia. This issue could receive better treatment in the discussion, and possibly even in the methods (produce adjusted pooled estimates). | We want to thank the reviewer for their comment. We do agree that this is an issue that needs to be addressed. We have addressed in detail in a previous response. In summary, we have pooled the most adjusted reported RRs, ORs, and HRs from the primary studies by using the inverse variance analysis with the random effects model. This is now reflected in the methodology, results, figures and abstract. However, given that we pooled aggregated data and that our studies are observational, we can only adjust for the variables the included studies adjusted for in their analyses. As such, we accept that there will some confounding in the results and thus we added the following in page 33 lines: 423-426 the discussion: “Also, we note that while we used the most adjusted results from the studies, we recognise that there is some degree of confounding which cannot be accounted for. This is because the included studies are observational and thus, we extracted aggregated data with variable degrees of adjustment.” |

**REVIEWER 2 COMMENTS**

| **Comments** | **Response** |
| --- | --- |
| Reviewer #2: See attachment  This paper presents a systematic review with meta–analysis of studies of  foetal loss and various maternal outcomes.  I was asked for a statistical report and I interpret that to include all aspects  of the design and conduct of the study. | We thank the reviewer for their time and effort in reviewing our work. We have carefully considered their suggestions and updated the manuscript as described below. |
| 1 Points of detail  Page 5 Just for the record there is now an updated PRISMA guideline (Page  et al., 2021a,b). I suspect any differences are fairly marginal. | We have now amended the manuscript to reflect the 2020 PRISMA guideline and added a supplementary figure with the relevant checklist (S2 fig). |
| Page 5 ‘The following [. . . ] were searched . . . ’. The actual database names  seem to have gone missing although they are listed in the abstract. | Thank you for highlighting. The databases were accidentally missed from the text in the methodology section. They have now been updated and the phrase on page 7 lines 107-109 reads: “Electronic databases including MEDLINE, EMBASE, AMED, BNI, CINAHL, and the Cochrane Library were searched from inception to June 2023 to identify studies exploring the association of fetal loss with subsequent maternal morbidity and mortality.” We have also included the detailed search strategy in the in supplementary figure 3. |
| Page 4 tells us that the majority of the cases of stillbirth are in low–  and middle–income countries. The existing search revealed few studies in LMICs. Various empirical studies have indicated that the main bibliographic databases have varied and incomplete coverage of material not in English (Pilkington et al., 2005; Shenderovich et al., 2016), and have limited coverage of material from low and middle–income country  journals (Kieling et al., 2009; Syed Sheriff et al., 2008). A wider search,  for instance in some of the WHO–sponsored ones, might throw up more  studies.  Page 5 and 6 The inclusion criteria (page 5) tell us that women with previous experience of loss were excluded but page 6 tells us it was a stratification variable. Does page 5 mean previous history of the outcomes not the types of loss? This needs clarifying. | We acknowledge that this can be misleading, and we have revised our statement in the introduction. Although we acknowledge that the majority of losses may happen in low- and middle-income countries, the majority of studies come from high income countries because data linkage and longer follow ups to enable estimation of long term morbidity and mortality are practically more feasible in high income countries. We still looked at the WHO sponsored websites and we were unable to retrieve studies reporting maternal disease.  We would like to thank the reviewer for highlighting this. We meant that studies in which women had a pre-existing diagnosis of the outcome of interest before experiencing a pregnancy loss were excluded. This is because we sought to examine the association of pregnancy loss with the incidence of long-term disease in women. We recognise that our phrasing was not clear and have amended the text on pages 8 lines 123-131: “Studies were included if they examined long-term incidence of cardiovascular, metabolic, malignant, mental health or any other morbidity in a population of women who had experienced fetal loss. Long-term was defined as disease developing at least a year after pregnancy loss. Additionally, we included studies investigating long-term all-cause mortality after pregnancy loss. Studies were excluded if patients had a history of previous diagnosis of disease outcomes described before experiencing pregnancy loss. Studies reporting maternal morbidity following a pregnancy termination were excluded from our review. Case reports, letters, in vitro and animal studies were also excluded, while we included cohort, case-control and cross-sectional studies were included.” |
| Page 7 I am not sure that using an arbitrary cut–off for heterogeneity is  helpful. As R ̈ucker et al. (2008) have argued it is quite misleading  when primary studies are large and give precise estimates. Many of  the studies included here had more than a quarter of a million women  enrolled which is large by most people’s standards. The discussion on  page 18 could also benefit from recognising this issue. | We would like to thank the reviewer for highlighting this. According to the citation provided, the authors concluded that Tau^2^ is the preferred method of assessing heterogenicity compared to I^2^. Our figures provide both results. We have now amended page 10 lines 167-168 the “statistical analysis” section in the methods to reflect this and it reads: “Heterogeneity was assessed using I^2^, Tau^2^ and Chi^2^ statistics.” In addition to this, a relevant amendment was made in the discussion on page 32, lines 417-421, and it reads: “It can be argued that while I^2^ is able to compare heterogenicity of meta-analysis with different numbers of studies, it has been shown to also increase in value with increasing sample size. In our meta-analysis some of the included studies involve many thousands of women and thus tau^2^ figures which is not affected by sample size, might be more useful in assessing heterogenicity.” |
| Page 9 Perhaps summarise the numbers per country to save the reader having to go through Table 1 and tot them up. The geographical spread is helpful to assess generalisability. | Thank you for your comment. The relevant sentence on page 11 lines 180-183 of the results section was amended to “Most studies were conducted in high-income countries including North America (n=12) Europe (n=35), Australia (n=2) and Israel (n=6). The remaining 4 studies were conducted in Asia (Japan: n=1, China: n=1, India: n=1, Iran: n=1,) including 1 high, 2 middle and 1 low-income countries.” |
| Page 9 Mean age at loss 23, mean age at follow up 69, mean follow up  period 16 years. 69 - 23 = 46. I can see the figures need not agree  exactly but that is a rather large discrepancy. | We have rechecked our data and calculations and we apologize for the mistake. The discrepancy arises from the difference in maternal age reported between case-control and cohort studies, with the former reporting age at outcome presentation and the latter age at recruitment (after pregnancy loss). Given the variability in the maternal age reported we decided to exclude this information from our results section. We have recalculated these figures and we now report the average follow-up in each of the arms of the study (miscarriage: 19 years, stillbirth:17 years). Page 11 lines 183-184 now reads: “The mean follow-up period for outcome assessment from index pregnancy for the miscarriage cohort was 19 while for stillbirth this was 17 years.” |
| Page 11 Not presenting a summary estimate is fine but we should still see the primary studies and a bit more detail about just why they differ so  much, the current text is rather cryptic. | We agree with the reviewer and have provided details on the primary included studies in systematic review in tables 1-2 and supplementary tables 1-4, so that an assessment on the differences between the studies can be made by the reader. |
| Page 12 The sentence starting ‘Moreover, endometrial’ seems to appear  twice. | The repeated sentence has been removed. |
| Page 15 I applaud the caution of stating first to our knowledge but the rest  of the discussion occasionally reverts to saying the first. | Thank you for highlighting this point. The discussion has now been amended to reflect this point. Specifically, the first sentence (page 29, lines 336-337) now reads: “We conducted a systematic review and meta-analysis examining the association between fetal loss and long-term maternal morbidity or mortality in women experiencing either miscarriage or stillbirth”. |
| Page 18 Finding few studies from LMIC seems to me worth mentioning as  a limitation here. | We agree with the reviewer on this point. This limitation has been added in the discussion on page 33, lines 428-429 it reads: “Lastly, most of the retrieved studies investigated the population from high-income countries, our findings may not be generalisable is middle- and low-income countries.” |
| Tables 1 and 2 Would these be easier to read if they were in landscape so  that more width could be given to some of the columns thus avoiding  having studies split over more than one page? | We have amended Table 1 and 2 in landscape format. |
| Forest plots The standard RevMan wording ‘favours stillbirth’ seems to  me rather unfortunate as having a higher risk of disease is hardly  favourable. | We want to thank the reviewer for this comment. We have now replaced it with exposure [increased risk] versus exposure [decreased risk] in the figures and supplementary information. |
| 2 Points of more substance  2.1 Some comments on the evidence base  It seems to me that there may be more going on in the datasets than is  currently presented. To illustrate this I took the first result mentioned in  the abstract and did some reanalysis on it. I modelled log risk ratios using  inverse variance weighting. I estimated τ2 using REML and used the Knapp–  Hartung adjustment (Knapp and Hartung, 2003). This has been shown to  give improved confidence interval coverage (Viechtbauer, 2005). I used the  R metafor package in version 3.8-1.  Rates in the control arm  The most striking feature is that the baseline rates vary in the non–stillbirth  arm. The largest rate is more than 300 times larger than the smallest. Even  if we ignore the two oldest studies on the basis that the world has moved  on since 1958 the largest is still more than 100 times the smallest. Having a  wide range of situations is obviously a good thing from the point of view of  generalisability but there must come a point when it becomes doubtful that  the same processes are at work in high incidence settings and low incidence  settings.  Prediction interval  Riley et al. (2011) have suggested that interpretation of random effects models should also include a prediction interval. I calculate that a 95% interval is from 0.82 to 3.76 on the risk ratio scale. In contrast to the confidence  interval which tells us about the precision with which we have estimated the  mean of the distribution of effects the prediction interval tells us where the  next study is likely to lie. In this case the prediction interval includes the  null value.  Confidence interval for heterogeneity  I estimate the 95% confidence interval for τ2 to stretch from 0.02 to 0.69 which  suggests that our estimates here must be regarded as rather uncertain. This  obviously affects a random effects model by introducing more uncertainty.  Would risk difference be more appropriate?  There are arguments both for and against using risk difference rather than  risk ratio. If we re–analyse using risk difference we find a central estimate of  0.019 with 95% confidence interval from -0.002 to 0.040 which obviousl (just)  includes the null value.  What conclusions would I draw?  My feeling from this re–analysis is that the conclusion should be that the  evidence base is not yet very strong. I have not examined any of the others.  None of this of course is the authors’ fault, we can only meta–analyse what  we can find, not what we would have liked to find. | We thank the reviewer for this detailed comment, and we agree with their conclusion that in meta-analysis we are limited to the data provided in the primary studies. We have taken various steps to mitigate some of the concerns raised:   1. By estimating ratios rather prevalence, the results are more likely to be representative of the magnitude of the existing associations and as rightly pointed more generalisable. 2. We have revised our analysis and conducted an inverse variance analysis where SE have been estimated and shown in the forest plots, so risk differences can be calculated. However, we feel the ratios are more clinically meaningful and easier to be explained to patients/ lay audience. 3. We estimated confidence intervals rather precision intervals as we want our results to be used in clinical settings showing the degree of uncertainty in our point estimates rather in research setting where prediction of a subsequent study estimates may be important.   We agree with the reviewer that the evidence may not be strong enough because of the degree of uncertainty, however, we still feel that we cannot ignore the results suggesting that reproductive exposures such as a history of stillbirth can facilitate earlier identification of women at higher risk of future disease. We have edited our conclusions in the discussion to show this more explicitly. |
| 2.2 Evidence of absence  In various places the authors seem to suggest that not being able to demonstrate an effect means that there is no effect. This is not the case and those instances would be better re–written as some form of ‘We were unable to demonstrate an effect. | We would like to thank the reviewer for noting this point. We have amended the text in many instances to reflect the suggested wording eg on page 29 lines 339-343 in the discussion to reflect this. It now reads: “We showed a 16% increase in the risk of T2DM after stillbirth. Additionally, women with a history of stillbirth are almost twice as likely to develop renal disease in the future. We were unable to demonstrate an association between history of stillbirth, single or recurrent miscarriage with future psychiatric disease, ovarian or any other female malignancies.” |
| 3 Summary  This is a report of a substantial piece of work. The length of my comments  should not be interpreted as a value judgment on it but as a justification of  my caution in accepting that the case is in any way closed. | We would like to thank the reviewer for dedicating time to peer-review our work. Given that the studies reporting these outcomes are observational, there is limitation to the extent that the meta-analysis methodology can be applied. We do appreciate this creates uncertainty with interpreting the results and we have highlighted this more clearly in the manuscript |

**REVIEWER 3 COMMENTS**

| **Comment** | **Response** |
| --- | --- |
| Reviewer #3: Thank you for the opportunity to review this interesting paper. The topic is important and the authors have gone to very considerable effort to consolidate and synthesise knowledge on this relatively overlooked area of research. I have a number of comments which may warrant consideration, particularly relating to the level of detail provided in the Methods section and how the Results are communicated. | We would like to thank the reviewer for the time and effort put into reviewing our work and we have carefully considered their suggestions and made changes as indicated in our responses. |
| Abstract:  Within the Methods of the Abstract, need to change "malignant" to "malignancy" | This has now changed as suggested. |
| Within Results section of the Abstract, it may be easier - or more intuitive - to present the results in the sequence of cardio/cerebro-vascular disease, renal disease, malignancy (results of which are counter-intuitive) and mental illness. | We agree with the reviewer’s point, and we have now amended the Findings Section of the Abstract on pages 3 lines 41-57 and it reads: “We included 56 studies, 45 of which were included in our meta-analysis. There were 1,119,815 women who experienced pregnancy loss of which 951,258 had a history of miscarriage and 168,557 experienced a stillbirth, compared with 11,965,574 women without a history of pregnancy loss.  Women with a history of stillbirth had a 56% (RR 1.56, 95% CI [1.30, 1.88]; p<0.001) greater risk of ischaemic heart disease (IHD), 71% greater risk of cerebrovascular (RR 1.71, 95% CI [1.44,2.03], p<0.001) and a 86% greater risk of any circulatory/cardiovascular disease (RR 1.86, 95% CI [1.01,3.45], p=0.05) compared with women without a history of stillbirth. We were unable to show an increased risk of cardiovascular disease (IHD: RR 1.11, 95% CI [0.98,1.27], or cerebrovascular: RR 1.01, 95% CI 0.85,1.21) in women experiencing a miscarriage.  Women with a history of stillbirth, were more likely to develop type 2 DM (RR: 1.17, 95% CI [1.07-2.26]; p<001). We were unable to show this in women experiencing a miscarriage. Women with a stillbirth history had double the risk of developing renal morbidities (RR 1.97, 95% CI [1.51,2.57], p<0.001) compared with women without. Women with a history of stillbirth had 20% (RR: 0.80, 95% CI [0.67,0.96], p-0.02) lower risk of breast cancer. We did not show an altered risk of other malignancies in women experiencing pregnancy loss compared to women without. We were unable to shaow a substantial risk of long-term mental illness in women with a history of pregnancy losses (stillbirth: (RR 1.90, 95% CI 0.93,3.88, miscarriage: RR 1.78, 95% CI 0.88,3.63).  We have also amended how we present our results. |
| Introduction:  Third paragraph is a little difficult to read. I think this could be phrased more convincingly, the rationale needs to be somewhat stronger. | We have now updated the introduction to make it easier to read and follow. We have also added a clearer rationale as to why our review was necessary. Page 5-6 lines 95-100 now read as follows:  Whether pregnancy is a “stress test” for long term disease, or whether common biological pathways related to circulation or inflammation underlie pregnancy losses and subsequent long-term disease is unclear. While existing data suggest an association between fetal loss and future subsequent morbidity, the literature is limited by small sample sizes and variation in study follow up. To address this need, we conducted a systematic review and meta-analysis to investigate the risk of long-term morbidity and mortality in mothers who experienced fetal loss of any type, including recurrent losses. |
| Methods:  In general, the Methods section is lacking in detail. Some of this may be due to word count consideration or the prior publication of a protocol on PROSPERO, but in its present state the Methods section does not provide sufficient detail for meaningful interpretation of the Results. | We would like to agree with the reviewer on this point. We initially reduced the details on the methodology of our study due to word count consideration, especially given that the protocol was previously submitted (and readily available) through PROSPERO. However, we do recognise that there are some important details missing and we have amended our methods section to reflect this. |
| The authors state that the grey literature was searched "by manually screening the reference list of papers included in the review and reviewing the reference list of related published systematic reviews". I am not convinced that this approach would provide a comprehensive overview of relevant grey literature and I would suggest rephrasing here. To my mind, this might add a very limited number of studies or technical reports which have not been peer reviewed, and which happen to have been cited by others previously. But it is unlikely to provide a broad, comprehensive scoping overview of the grey literature. | We have now completed a more comprehensive review of the grey literature and specifically looked at the System for Information on Grey Literature (OpenSIGLE), Healthcare Management Information Consortium (HMIC) database, National Technical Information Service (NTIS) and PsychEXTRA and we did not find any additional reports looking at pregnancy losses and development of long-term disease. We have updated the methodology to reflect this. Page 7, lines 117-119 now reads: “Additionally, we searched the System for Information on Grey Literature (OpenSIGLE), Healthcare Management Information Consortium (HMIC) database, National Technical Information Service (NTIS) and PsychEXTRA for relevant citations.” |
| There is insufficient detail given on what outcomes the authors sought, and how these were ascertained. For example, in terms of neoplastic outcomes, were benign tumours included? If so, were diagnoses of benign tumours potentially included within the same meta-analyses as mortality from metastatic cancer? (And if so, this would likely introduce an unacceptable degree of clinical diversity in to meta-analyses) How were decisions taken to differentiate outcome measures? Within the Results section it becomes more apparent that the authors focused particularly on breast, ovarian, endometrial and cervical cancer, but this is not clear from the Methods.  Further detail is required on methods of outcome ascertainment. Did the authors accept any self-reported diagnosis of an outcome, or was a doctor's diagnosis or laboratory/histological confirmation required? Or did they rely on ICD/DSM coding of relevant diagnoses? This detail is required for comparison with any future studies. If multiple methods of outcome ascertainment were accepted, ideally subgroup analyses might be conducted with this differentiation in mind. | We acknowledge that meta-analysis is limited to the data provided in the primary studies. We had published in our protocol that we would include all outcomes and we did not want to exclude studies reporting self-reported data as this would introduce selection bias. We have updated our tables with the addition of supplementary tables 1 and 2 to include how outcomes were defined in each so the readers can use them as reference. In regard to malignancies, we did not select studies that reported benign tumours. We feel that including further subgroup analysis in the supplementary material will be confusing, but we have analysed the studies providing ICD/DSM coding for outcomes and the results have not materially changed. We can provide these analyses if the reviewer feels strongly that needs to be included in the revised manuscript. |
| Results:  In Figure 1 it is stated that there were 1080 "additional records identified through other sources". I am not clear on what is meant by this. Are these all from reference lists of other articles? This seems like a very large number of articles to add to the flow diagram without a full explanation for their source. | We want to thank Reviewer for highlighting this point. The denotation of “additional records identified through other sources” was intended to describe records obtained and subsequently screened from the reference lists of the included studies. We have made this clearer in the updated PRISMA flowchart (fig 1). |
| The second paragraph of Results section is problematic for a few reasons:  "The mean maternal age at the time of fetal loss was on average 23 years at the time of loss (Pell et al., 2004) and 69 years (Parker et al., 2014) at the time of outcome measure." Firstly, the study by Pell et al. (2004) is not within the list of References. Secondly, it is unclear from this sentence how these references should be applied here? Is the mean maternal age at the time of fetal loss not based on the entire pooled cohort of affected women across all eligible studies, and likewise for the age of outcome measure? If so, why would only one reference apply for each of these points? Moreover, the next sentence states "The mean follow-up period for outcome assessment for the miscarriage cohort was 16.9 while for stillbirth this was 16.4 years." If this is true, then one would imagine that the mean age at outcome measurement would be approximately 16-17 years after their most recent fetal loss (but perhaps not exactly so, depending on which studies reported this data).  Based on this rationale, one might expect that women were, on average, aged 52-53 years at their most recent fetal loss (i.e. 69-16 = age 53 years) according to the description provided. Presumably this is incorrect. Maybe I have misinterpreted what the authors are trying to communicate here - but if so, I think there is scope for others to misinterpret this too. I think the phrasing needs to be improved throughout this paragraph. | We would like to thank Reviewer for highlighting this valid point. The initial statement intended to report the average maternal age across all cohorts in the included studies at the time of pregnancy loss. Similarly, the second part of the sentence intended to report the average maternal age at outcome presentation. We have rechecked our data and calculations and we apologize for the mistake. The discrepancy arises from the difference in maternal age reported between case-control and cohort studies, with the former reporting age at outcome presentation and the latter age at recruitment (after pregnancy loss). Given the variability in the maternal age reported we decided to exclude this information from our results section to avoid misunderstanding and confusion. This was also done as some of the studies did not report data on maternal age and thus the mean age from our data would not be representative of the true value. We have, however, recalculated the average follow up time in each of the arms of the study (miscarriage: 19 years and stillbirth: 17 years). The relevant sentence in the first paragraph of the results on page 11 lines 183-184 now reads: “The mean follow-up period for outcome assessment for the miscarriage cohort was 19 while for stillbirth this was 17 years.” |
| Outcomes:  It is difficult to know how to interpret some of these findings without further detail on how the authors dealt with the issue of pre-existing maternal morbidity. Were all analyses based on women who had no prior diagnosis of the outcome in question (e.g. for IHD, were all women with pre-existing cardiovascular disease excluded at baseline? What about major risk factors for IHD e.g. pre-existing chronic hypertension?) I think some subgroup analysis is required for some of the key results. For example, in figure 2ai, the largest studies all appear to show a statistically significant association between miscarriage and IHD, but there are multiple smaller studies which do not. The forest plot suggests a modest, but non-significant association overall, and the authors present this as a null association. I am unsure about this interpretation. Could it be an issue related to methodological diversity between included studies in the meta-analysis (based on sample size, or study design)? A similar issue arises for figure 2bi where the largest studies of miscarriage and stroke all appear to show a statistically significant association (perhaps based on greater statistical power?) but multiple smaller studies show a null association, and this is also what the forest plot concludes. I think subgroup analysis could help to explain whether true associations might exist or not. | We excluded studies that the outcome of interest was diagnosed before the pregnancy loss, because we were interested in looking at the association of the exposure with the incidence of the disease. In addition, we excluded studies that looked at outcomes in < 1 year from exposure to minimise the possibility of reverse causality. We have updated our analysis by pooling the most adjusted ratios and used a random effects model to mitigate some of the between studies. We could do the subgroup analysis suggested, but we feel that this will not be clinically meaningful or entirely justifiable based on study size only. However, we can include this if the reviewer feels strongly about it. |
| Minor point, on page 12 the sentence "Moreover, endometrial (Mikkelsen et al., 2019) (Xu et al., 2004) and ovarian (Mikkelsen et al., 2019) malignancy risk was examined in two and one studies respectively, while cervical malignancy risk was assessed one paper (Mikkelsen et al., 2019)." appears twice. | The repeated phrase has now been removed. |
| The findings for Mikkelsen 2019 are counter-intuitive and seem to heavily influence the results of the meta-analysis of stillbirth and breast cancer (which feature prominently, including in the Abstract) and which is ultimately based on a small number of included studies (n=3). From a quick look at the original analysis by Mikkelsen et al. 2019, their main analysis reported a null association between stillbirth and breast cancer (rather than a seemingly protective effect). Perhaps the authors chose to compare number of outcomes among parous women only - and if so that's fine. But it would be helpful to state whether this approach was consistently applied across all studies (if so, this needs to be clearer in the Methods section). When the authors use the term "controls", do they mean parous women who never experienced pregnancy loss? Or do they mean all women (including those who were never pregnant) who have never experienced pregnancy loss? This distinction is important for the interpretation of the Mikkelsen 2019 data. I would also suggest making it clear that this result is based on a small number of studies and is very much dominated by the authors' interpretation of the data presented by Mikkelsen et al., otherwise there may be scope for misinterpretation of the clinical relevance of this.  Regarding mental health outcomes, these results are also difficult to interpret without some detail on outcome ascertainment and definitions of various outcomes (e.g. depression). Clearly, the meta-analysis findings are approaching statistical significance and thus should be interpreted with some caution. Again, is there scope for some subgroup analysis to aid interpretation here? | We acknowledge that the number of the studies looking at the association with breast cancer is small and we have made it clear that our controls is all women (parous and nulliparous) without a pregnancy loss. We have also discussed the small number of studies for breast cancer and the need for replicating the results as a limitation in the discussion. The relevant section in our discussion on page 31 lines 387-389 reads: “The mechanisms mediating the modest risk reduction for history of stillbirth warrant further laboratory, clinical and epidemiological investigations but may be partially related with reduced gestational duration and therefore exposure to high levels of reproductive hormones. A well-defined comparator group and good phenotype of breast cancer types may shed more light on the mechanisms underlying this association. Additionally, our analysis involved a small number of studies, something necessitating further research with replication of results in future studies.”  We have now added supplementary tables 1 and 2 to include how outcomes were defined in each study. We feel that further subgroup analysis will lead to less clear conclusions because of loss in power. However, if the reviewer feels strongly about it we can include this in the revised version. |
| Discussion:  Several of the aforementioned points need to be addressed either within the body of the manuscript or within a much more lengthy section on Limitations.  The comparison of findings between this manuscript and the previous systematic review by Oliver-Williams et al. requires further elaboration. Are there other methodological differences which existed between these two systematic reviews?  It would be helpful to have some further discussion on what the authors believe to be the implications of their findings, including consideration of the absolute risks to affected women if associations herein are true. Miscarriage is considerably more prevalent than stillbirth, and thus associations between stillbirth and maternal morbidity may be important in relative terms, but less concerning in absolute terms compared to even modest associations between miscarriage and maternal outcome measures. | We agree with the reviewer ‘s comment. We have the relevant paragraph in the discussion section on pages 32-33 lines 413-428 of the manuscript to include the limitations of the study. This paragraph now reads: “However, there are also some limitations. Firstly, the heterogeneity in some of our results was relatively high for both the stillbirth and miscarriage groups. This heterogeneity could be explained by the small number of studies, which might not be fully representative of the prevalence of these conditions in the general population. It can be argued that while I^2^ is able to compare heterogenicity of meta-analysis with different numbers of studies, it has been shown to also increase in value with increasing sample size. In our meta-analysis some of the included studies involve many thousands of women and thus tau^2^ Figures which is unaffected by sample size, might be more useful in assessing heterogenicity. [86] Additionally, the follow-up period, sample size reported, as well as definitions of stillbirth and miscarriage also varied between studies, further contributing to this variation. Also, we note that while we used the most adjusted results from the studies, we recognise that there is some degree of confounding which cannot be accounted for. This is because the included studies are observational and thus, we extracted aggregated data with variable degrees of adjustment. Some of the reported outcomes, for example breast cancer in the stillbirth arm involved a small number of studies, something which warrants further research to investigate reliability of results. Lastly, most of the retrieved studies investigated the population from high-income countries, our findings may not be generalisable is middle- and low-income countries”  Regarding the differences with the review by Oliver-Williams et al when we compared our forest plots, there is evidence that with have included more contemporary and larger (more weight on meta-analysis) primary studies which had shown no significant association and thereby pulled the pooled estimate towards the null effect. We have included this in the discussion.  We have also elaborated further in the conclusion the clinical implications of our findings. |

References

Kyriacou, H., Al-Mohammad, A., Muehlschlegel, C., Foster-Davies, L., Bruco, M.E.F., Legard, C., Fisher, G., Simmons-Jones, F. and Oliver-Williams, C., 2022. The risk of cardiovascular diseases after miscarriage, stillbirth, and induced abortion: a systematic review and meta-analysis. *European Heart Journal Open*, *2*(5), p.oeac065.
