# Supplementary material for: Fetal loss and long-term maternal morbidity and mortality: A systematic review and meta-analysis
Source: PLoS Med. 2024 Feb 9;21(2):e1004342. doi: 10.1371/journal.pmed.1004342 (PMC10857720; doi:10.1371/journal.pmed.1004342)
Supplement: Attachment — Submitted filename: Response letter.docx [file pmed.1004342.s005.docx]

**Response letter** (PMEDICINE-D-23-00438R2)  Decision - [EMID:ecdf2678ece1eb22]

Manuscript title: Fetal loss and long-term maternal morbidity and mortality: a systematic review and meta-analysis

Dear Dr Schaefer,

Many thanks to the editorial team and the reviewers for the time and effort in evaluating the manuscript changes and for the provisional acceptance of our manuscript. We have now addressed the new comments individually and edited the manuscript accordingly.

Best wishes,

Jan Daru, on behalf of all the co-authors

**ACADEMIC EDITOR COMMENTS**

| **Comments** | **Response** |
| --- | --- |
| The authors have tried to address the issues as well as they can - however, I agree with the reviewer that the conclusions remain overstated, given the limitations of the study (albeit that limitations are now discussed more fully). | We appreciate the comment by the academic editor, and we have toned down the conclusions in the discussion. We specifically added lines 394-401 to address the fact that risk factors for stillbirth such as pre-eclampsia and gestational diabetes are well established risk factors for CVD and that the confounding effect of this on our results. Lines 394-401 now read: “However, we acknowledge that risk factors for stillbirth such as pre-eclampsia and gestational diabetes are established risk factors for CVD. Given the available data in aggregated studies, we were unable determine the impact of these factors individually in this review. It is possible that data on combined risk factors (such as gestational diabetes and preeclampsia combined, along with others) may have influenced our findings. Our results suggest that a of history of stillbirth maybe an indicator of cardiovascular risk over and above established risk factors such as gestational diabetes for long term morbidity, and merits further research.” This is also reflected in lines 416-417 and lines 494 to 496 in the conclusion. |

**EDITORIAL COMMENTS**

| **Comments** | **Response** |
| --- | --- |
| In line with the comments made by the Academic Editor and Reviewer #1, we suggest toning down the conclusions throughout the paper. | We appreciate the comment by the academic editor, and editing team and we have toned down the conclusions stated in the discussion. We have toned down the conclusions in the discussion. We specifically added lines 394-401 to address the fact that risk factors for stillbirth such as pre-eclampsia and gestational diabetes are well established risk factors for CVD and that the confounding effect of this on our results. Lines 394-401 now read: “However, we acknowledge that risk factors for stillbirth such as pre-eclampsia and gestational diabetes are established risk factors for CVD. Given the available data in aggregated studies, we were unable determine the impact of these factors individually in this review. It is possible that data on combined risk factors (such as gestational diabetes and preeclampsia combined, along with others) may have influenced our findings. Our results suggest that a of history of stillbirth maybe an indicator of cardiovascular risk over and above established risk factors such as gestational diabetes for long term morbidity, and merits further research.” This is also reflected in lines 416-417 and lines 494 to 496 in the conclusion. |
| Similarly, in line with Reviewer #1 comments, we feel that that the influence of comorbidity should be better discussed/disentangled, particularly with regard to the potential for gestational diabetes and preeclampsia, both of which predispose to both stillbirth and CVD. We agree that this merits additional discussion in the paper regarding these potentially explanatory pathways with respect to CVD outcomes | We appreciate the comment by Reviewer 1, and editing team and we have toned down the conclusions stated in the discussion. We specifically added lines 394-401 to address the fact that risk factors for stillbirth such as pre-eclampsia and gestational diabetes are well established risk factors for CVD and that the confounding effect of this on our results. Lines 394-401 now read: “However, we acknowledge that risk factors for stillbirth such as pre-eclampsia and gestational diabetes are established risk factors for CVD. Given the available data in aggregated studies, we were unable determine the impact of these factors individually in this review. It is possible that data on combined risk factors (such as gestational diabetes and preeclampsia combined, along with others) may have influenced our findings. Our results suggest that a of history of stillbirth maybe an indicator of cardiovascular risk over and above established risk factors such as gestational diabetes for long term morbidity, and merits further research.” This is also reflected in lines 416-417 and lines 494 to 496 in the conclusion. |
| We concur with the statistical reviewer's suggestion to include prediction intervals alongside confidence intervals in the manuscript. Kindly integrate prediction intervals into your analysis. | We understand the merit in adding prediction intervals in the interpretation of results. Thus, we calculated the prediction intervals using Python with the following code: <https://bit.ly/flo-plosone-intervals-python>. The abstract (lines 20-74), methodology (page 12, lines 213-215) and results sections have been edited to include the above information and values are presented in the following format: 95% PI [x,y]. |
| The studies included are all observational - please state this in the abstract (also see comment 1) under ABSTRACT). | Thank you for highlighting. Page 3 line 41 of the Methods and Findings section of the Abstract now reads: “We included 56 observational studies, 45 of which were included in our meta-analysis.” |
| In the title, please exchange the semicolon with a colon. | This has been exchanged and the title on page 1, line 2 reads: “Fetal loss and long-term maternal morbidity and mortality: a systematic review and meta-analysis.” |
| Regarding presentation style (e.g., Abstract 'Findings section' or Discussion), we ask that you not write, e.g., "...had a 56% greater risk (RR 1.56 ...); "had a greater risk (RR 1.56 ..." is our preferred style. Similarly, phrases like "we were unable to show" should be exchanged with "there was no evidence of increased risk" or similar. | Thank you for noting. The Methods and Findings section of the Abstract was edited to read as (page 3, lines 44-58): “Women with a history of stillbirth had a greater risk of ischaemic heart disease IHD (RR 1.56, 95% CI [1.30, 1.88]; p<0.001)), cerebrovascular (RR 1.71, 95% CI [1.44,2.03], p<0.001), and any circulatory/cardiovascular disease (RR 1.86, 95% CI [1.01,3.45], p=0.05) compared with women without a history of stillbirth. There was no evidence of increased risk of cardiovascular disease (IHD: RR 1.11, 95% CI [0.98,1.27], or cerebrovascular: RR 1.01, 95% CI 0.85,1.21) in women experiencing a miscarriage.  Women with a history of stillbirth, were more likely to develop type 2 DM (RR: 1.17, 95% CI [1.07-2.26]; p<001). There was no evidence of this in women experiencing a miscarriage. Women with a stillbirth history had an increased risk of developing renal morbidities (RR 1.97, 95% CI [1.51,2.57], p<0.001) compared with women without. Women with a history of stillbirth had lower risk of breast cancer (RR: 0.80, 95% CI [0.67,0.96], p-0.02). There was no evidence of an altered risk of other malignancies in women experiencing pregnancy loss compared to women without. There was no evidence of a substantial risk of long-term mental illness in women with a history of pregnancy losses (stillbirth: (RR 1.90, 95% CI 0.93,3.88, miscarriage: RR 1.78, 95% CI 0.88,3.63). |
| When revising your manuscript, please check carefully for punctuation. | We have checked the manuscript’s punctuation. |
| **ABSTRACT** |  |
| Thank you for explaining that details about the types of study designs included, and synthesis/appraisal methods are stated within the Methods sections. Please also include these details in the Abstract. | The Methods and Findings section of the Abstract has now been amended to include more details on the data synthesis / appraisal methods used. |
| Please combine the Methods section and Findings section into one Methods and Findings section. | The two sections have now been combined as suggested. |
| l.51: Please fix "p<001". | This has now been changed to "p<0.001”. |
| l.57: Please include square/box brackets for the CI values. | CI values are now enclosed in square brackets throughout the manuscript. |
| In the last sentence of the Abstract Methods and Findings section, please describe the main limitation(s) of the study's methodology. | We added the following sentence on page 4, lines 62-63 to describe our study’s main limitation: “The study’s main limitation includes the potential for confounding as we extracted aggregated data with variable degrees of adjustment”. |
| **AUTHOR SUMMARY** |  |
| Thank you for providing an Author Summary. Unfortunately, the current Author Summary does not conform to our requirements. The summary should include 2-3 individual bullet points (single sentences for each) under each of the questions (Why Was This Study Done?; What Did the Researchers Do and Find?; What Do These Findings Mean?). In the final bullet point of 'What Do These Findings Mean?', please describe the main limitations of the study in non-technical language. Please see our author guidelines for more information: <https://journals.plos.org/plosmedicine/s/revising-your-manuscript#loc-author-summary>. It may be helpful to review currently published articles for examples which can be found on our website here <https://journals.plos.org/plosmedicine/> | Thank you for highlighting. The Author Summary has been edited to reflect the journal’s requirements and page 4-5, lines 72-95, it now reads:  ” **Why was this study done?**   - Fetal loss is one of the most devastating complications of pregnancy, with approximately 23,000,000 miscarriages and 2,000,000 stillbirths reported globally per annum. - Evidence suggests that there is relationship between pregnancy loss and subsequent development of maternal disease. - Given this, we conducted a systematic review and meta-analysis on the clinical evidence available to better understand the effect of pregnancy loss on the long-term maternal morbidity and mortality across all body systems.   **What did the researchers do and find?**   - Our systematic review included a total of 56 observational studies, with a total of 1,119,815 (miscarriage: 951,258, stillbirth: 168,557) women who experienced pregnancy loss and 11,965,574 controls. - Women with a history of stillbirth were found to have an increased risk of developing ischemic heart, cerebrovascular, circulatory and renal disease, and type 2 diabetes mellitus as well as a slight decrease in the risk of breast cancer. - There was no evidence of increased risk of developing these conditions in the miscarriage arm.   **What do these findings mean?**   - Our results suggest a possible association of stillbirth with future cardiovascular disease, type 2 diabetes and renal disease, signifying that pregnancy loss could guide risk and lifestyle management. - However, given that we extracted aggregated data with variable degrees of adjustment, the potential for confounding variables such as pre-eclampsia and gestational diabetes (both of which as well-known risk factors for metabolic syndrome) affecting the results is considerable. - Thus, further research accounting for these variables is necessary to ascertain the true relationship of pregnancy loss and future maternal disease.” |
| **INTRODUCTION** |  |
| l.81: Please define 'ICD' at first use. | This has now been defined and line on page now reads: “in accordance with the ICD-10 (International Classification of Diseases) criteria (ICD-10)” |
| l.86: The first quotation marks are missing, please add. | This has now been added. |
| l.89: "Stillbirth is reported in more than 2,500,000 women annually." - please add a reference. | The reference has been added and the figure was amended to reflect WHO’s epidemiological statistics and it now reads (page: 5, lines 90-91): “Stillbirth is reported in approximately 2,000,000 women annually [1].” |
| ll.180-182: This sentence currently reads as if North America and Europe are high-income countries. Please re-phrase (e.g., 'high-income regions/countries...'). | Thank you for highlighting. Line 183 on page 11 now reads: “studies were conducted in high-income countries / regions including North America (n=12) Europe (n=35),”. |
| l.189: "(37/59)" - please change to, e.g. (n=37 out of 59) or (n=37). | The text on page 11 line 188 now reads: “(37 out of 39)”. |
| Table 1: In the column 'Duration of follow-up', some numbers do not have units (years?, months?) - please revise. | Thank you for pointing this out. All figures in this column now have the appropriate time unit next to the number. |
| Table 1: In the 'Result' column in brackets, one definition next to RR, OR etc. seems to be missing. For example, for the study of Andalib et al. (2006), Iran, the results seem to be presented as "n ± SD". | Thank you for highlighting. The measures missing are the Beck Depression Index and the Life Change Units which measure depression and stress respectively. These have been added to the heading of the column, on the relevant rows in table 1 (Andalip and Bergant), and on the abbreviation list under the table. |
| Table 1: Please ensure that for all numbers in the 'Result' column it is evident what the numbers are (RR, OR etc.). For example, for Bergant et al. (1997), Austria, it is not clear. Also, when presenting 95% CI values, please ensure to add "95% CI" before the brackets. | All the numbers have been labelled and accounted for table 1. |
| Table 1 and Table 2: Please note the use of commas to separate upper and lower bounds, as opposed to hyphens as these can be confused with reporting of negative values. | The hyphens have now been replaced in both tables 1 and 2. |
| Table 1: When stating age, please add a unit (e.g., for Toffol, Koponen, Partonen (a) (2013), Finland). | This has been added for all missing time units. |
| Table 1: Please define TIA. | TIA has been defined as “Transient Ischaemic Attack” in the abbreviation section under table 1. |
| Table 2: Please define HR. | HR has been defined as “Hazard Ratio” in the abbreviation section under table 2. |
| Table 2: When presenting 95% CI values, please ensure to add "95% CI" before the brackets | The phrase “95% CI” has been added in every relevant figure on table 2 in the Results section. |
| l.241: Is IHD correct here as the section discusses cerebrovascular disease including haemorrhagic and ischaemic stroke? | “IHD” has now been replaced with “cerebrovascular disease” on page 26 line 244. |
| l.271: "[11,31,35]" seems to be misplaced - please revise. | The references have now been moved. |
| l.288: "...without a history, of which 6,381 (2.78%) exposed and 23,514 (3.12%)" - The sentence seems to be incomplete, please revise. | The sentence has now been edited and it reads (page 28-29, lines: 329-331): “Data were extracted from all studies but Goldacre et al (2001) (provided no extractable data) and included 229,606 women with a history of miscarriage and 753,236 without a history, of which 6,381 (2.78%) and 23,514 (3.12%) developed breast cancer respectively. “ |
| l.299: "[20,44,57,60]" seems to be misplaced - please revise the placement of references through the entire main manuscript. | The references have now been updated. |
| **DISCUSSION** |  |
| ll.393-395: Please provide references. | The references have been added to line 450. |
| Please remove the 'Conclusion' subheading. The 'Conclusion' paragraph should be the last part of the Discussion section. | The subsection has been removed and the “conclusion” paragraph is the last part of the discussion. |
| FIGURES |  |
| The Figures are of very low quality, please check. | Thank you for highlighting. We have tried to enhance the quality of the figures, and these have been uploaded separately as per journal requirements. |
| Figure 1: In the 'Records identified from:' box, please name the specific databases with the according numbers of records identified. | The specific databases were added on the relevant section of the figure and the updated figure has been uploaded. |
| Figure 2: In the Figure description, please add "versus" to "(bii) Cerebrovascular in women with stillbirths no stillbirth". | The word “vs” was added in figure 2 as suggested. |
| Figure 2 and Figure 3: Please revise the x-axis labels as it appears that on some graphs 'Exposure [Increase Risk]' and 'Exposure [Decrease Risk]' are on the wrong side. | All x-axis labels have been revised to reflect the correct denotation. |
| Figure 2/Figure 3/Figure 4/Figure 5: Please delete the "year" column and only add the year to first column 'Study or Subgroup'. | The column “Year” has been removed from all figures. |
| SUPPLEMENTARY MATERIALS |  |
| Please provide the completed PRISMA checklist and the Abstract PRISMA checklist as separate Supporting Information files and do not list them as S Figures. | The PRISMA Checklists for both the Abstract and the Manuscript have been removed from the existing file and a separate supporting information file has been created and will be submitted. These checklists were removed from the S figures in the manuscript and the references to text were edited accordingly. |
| Please ensure that you provide x-axis labels for all graphs (e.g. see S8 Fig (c)). | Thank you for noting. X-axis labels have been added in all of the supplementary figures including a forest plot. |
| S8 Fig - S17 Fig: Please delete the "year" column and only add the year to first column 'Study or Subgroup'. | The “Year” column has been removed from all supplementary figures as recommended. |
| Please ensure to define all abbreviations used in your supplementary figures and tables. | All abbreviations have been defined in the supporting information. |
| REFERENCES |  |
| Where website addresses are cited, please use 'accessed' instead of 'cited' when specifying the date of access. | The word “cited” has been replaced with “accessed” when referencing the date of access in the entirety of the reference list. |
| Please thoroughly revise all references and ensure that journal name abbreviations match those found in the National Center for Biotechnology Information (NCBI) databases (<http://www.ncbi.nlm.nih.gov/nlmcatalog/journals>), and are appropriately formatted and capitalised (e.g., for reference [17] American Journal of Obstetrics and Gynecology should be Am J Obstet Gynecol). | All references have been thoroughly revised to reflect the journal’s guidelines, including the NCBI abbreviations. |
| SOCIAL MEDIA |  |
| To help us extend the reach of your research, please provide any X (formerly known as Twitter) handle(s) that would be appropriate to tag, including your own, your coauthors', your institution, funder, or lab. Please respond to this email with any handles you wish to be included when we tweet this paper. | Please use the following X handles:  @CphpQmul  @QMUL_WIPH  @JDaru  @M_Iliodromiti |

**COMMENTS FROM REVIEWERS**

**Reviewer 1**

| **Comments** | **Response** |
| --- | --- |
| I think that the revised version has improved. I selected "Proceed without recommendation" because I felt the overall conclusion was somewhat overstated. The problem is that it is very well known that gestational diabetes and preeclampsia are strong determinants of CVD. These pregnancy disorders are also strongly associated with stillbirth.  Reading the discussion, it felt like not enough emphasis was put on these potential explanatory pathways (guidelines for CVD risk reduction include these two pregnancy conditions, so an argument for including stillbirth seems uncertain).  However, the manuscript is also fine in this form if the editors are satisfied with the revision. Thank you for consulting me. | We thank Reviewer 1 for their suggestions. We agree that conditions such as pre-eclampsia and gestational diabetes in women with a history of stillbirth, which are well established risk factors of CVD have the potential of introducing bias in the results. Thus, we have tried to tone down the conclusions stated in the discussion. We specifically added lines 401-418 to address the fact that risk factors for stillbirth such as pre-eclampsia and gestational diabetes are well established risk factors for CVD and that the confounding effect of this on our results. Lines 401-408 now read: “However, we acknowledge that risk factors for stillbirth such as pre-eclampsia and gestational diabetes are established risk factors for CVD. Given the available data in aggregated studies, we were unable determine the impact of these factors individually in this review. It is possible that data on combined risk factors (such as gestational diabetes and preeclampsia combined, along with others) may have influenced our findings. Our results suggest that a of history of stillbirth maybe an indicator of cardiovascular risk over and above established risk factors such as gestational diabetes for long term morbidity, and merits further research.” This is also reflected by lines 489-491 in the limitation section of the discussion and lines 501-503 in the conclusion. |

**Reviewer 2**

| **Comments** | **Response** |
| --- | --- |
| The authors' rebuttal is fine but contains one statement which I find hard to accept.  I recommended also presenting prediction intervals as well as confidence intervals. The authors replied that "... we want our results to be used in clinical settings showing the degree of uncertainty in our point estimates rather in research setting where prediction of a subsequent study estimates may be important."  If a group of clinicians reads this review and asks themselves "What would happen here?" then that is exactly the same as asking what would happen in the next study and so for that purpose they need a prediction interval. Knowing the variance of the distribution of true effect sizes is a theoretical interest. So my feeling is that the authors have this the wrong way round. | We want to thank Reviewer 2 for highlighting this point. We understand the merit in adding prediction intervals in the interpretation of results. Thus, we calculated the prediction intervals using Python with the following code: <https://bit.ly/flo-plosone-intervals-python>. The abstract, methodology and results sections have been edited to included the above information and values are presented in the following format: 95% PI [x,y]. |

**Reviewer 3**

| **Comments** | **Response** |
| --- | --- |
| I am satisfied that the authors have addressed my queries. Thank you for considering my previous feedback and for the opportunity to review this relevant and timely article. | We would like to thank Reviewer 3 for their time and effort in reviewing our paper. |
